# Genomic Landscape of Intramedullary Spinal Cord Gliomas

**DOI:** 10.1038/s41598-019-54286-9

**Published:** 2019-12-10

**Authors:** Ming Zhang, Rajiv R. Iyer, Tej D. Azad, Qing Wang, Tomas Garzon-Muvdi, Joanna Wang, Ann Liu, Peter Burger, Charles Eberhart, Fausto J. Rodriguez, Daniel M. Sciubba, Jean-Paul Wolinsky, Ziya Gokaslan, Mari L. Groves, George I. Jallo, Chetan Bettegowda

**Affiliations:** 1 Ludwig Center for Cancer Genetics, Sidney Kimmel Cancer Center, Johns Hopkins University School of Medicine, Baltimore, MD 21287 USA; 2 Department of Neurosurgery, Johns Hopkins University School of Medicine, Baltimore, MD 21287 USA; 3 Stanford University School of Medicine, Stanford University, Stanford, CA USA; 4 Present Address: Department of Neurological Surgery, UT Southwestern Medical Center, Dallas, TX USA; 5 Department of Medicine, Johns Hopkins University School of Medicine, Baltimore, MD 21287 USA; 6 Department of Pathology, Johns Hopkins University School of Medicine, Baltimore, MD 21287 USA; 7 Present Address: Department of Neurosurgery, Northwestern University School of Medicine, Chicago, IL USA; 8 Present Address: Department of Neurosurgery, Brown University School of Medicine, Providence, RI USA; 9 Present Address: Department of Neurosurgery, Johns Hopkins All Children’s Hospital, Saint Petersburg, FL USA

**Keywords:** CNS cancer, Cancer genomics

## Abstract

Intramedullary spinal cord tumors (IMSCTs) are rare neoplasms that have limited treatment options and are associated with high rates of morbidity and mortality. To better understand the genetic basis of these tumors we performed whole exome sequencing on 45 tumors and matched germline DNA, including twenty-nine spinal cord ependymomas and sixteen astrocytomas. Though recurrent somatic mutations in IMSCTs were rare, we identified *NF2* mutations in 15.7% of tumors (ependymoma, N = 7; astrocytoma, N = 1), RP1 mutations in 5.9% of tumors (ependymoma, N = 3), and ESX1 mutations in 5.9% of tumors (ependymoma, N = 3). We further identified copy number amplifications in *CTU1* in 25% of myxopapillary ependymomas. Given the paucity of somatic driver mutations, we further performed whole-genome sequencing of 12 tumors (ependymoma, N = 9; astrocytoma, N = 3). Overall, we observed that IMSCTs with intracranial histologic counterparts (e.g. glioblastoma) did not harbor the canonical mutations associated with their intracranial counterparts. Our findings suggest that the origin of IMSCTs may be distinct from tumors arising within other compartments of the central nervous system and provides the framework to begin more biologically based therapeutic strategies.

## Introduction

Intramedullary spinal cord tumors (IMSCTs) are a heterogeneous group of rare lesions that can cause significant morbidity in both children and adults. These tumors are predominantly glial in origin, account for approximately 10–20% of all spinal tumors, and occur with less frequency than their histopathologic intracranial counterparts^1,2^. The optimal treatment of IMSCTs is maximal safe surgical resection, with adjuvant chemotherapy and radiation therapy (RT) playing a role in recurrent or progressive residual disease, as well as cases in which morbidity associated with surgical intervention is thought to be too high^3,4^.

Identification of the pathophysiological mechanisms underlying IMSCT development has been challenging because of the rarity of these tumors and heterogeneity amongst tumor subtypes. Investigation of inheritable syndromes such as neurofibromatosis type 1 and 2 (NF1, NF2) and von-Hippel Lindau (vHL) has illuminated particular genetic aberrations that can lead to IMSCT formation; however, these molecular mechanisms are often absent in sporadic IMSCTs, whose genetic and molecular profiles are less well understood^5–9^. Strategies for chemotherapeutic intervention for IMSCTs have included the application of established regimens and protocols described for other central nervous system (CNS) tumors with similar histology^10–13^. Although clinically appealing, this approach may be ineffective and potentially hazardous given an increasing body of evidence that the genetic makeup and mutation spectra of histologically comparable CNS tumors can vary considerably^14–18^.

An overall dearth of literature exists regarding specific genetic alterations in IMSCTs^9,14,19–22^. Improved understanding of the molecular pathophysiology of these tumors could lead to more effective therapeutic strategies. Therefore, we performed whole-exome sequencing (WES) and whole-genome sequencing (WGS) of a diverse cohort of IMSCTs to elucidate the genetic profiles of IMSCT subtypes.

## Results

### Cohort characteristics

**Figure 1:**
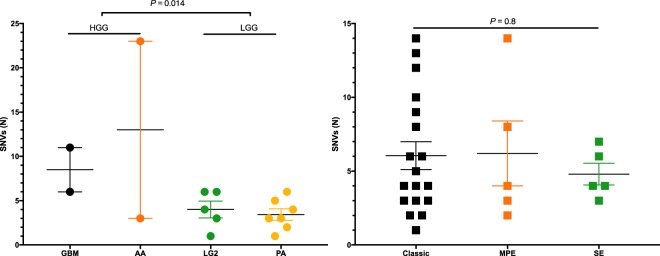
Whole exome mutational burden of intramedullary spinal cord tumors. (**A**) astrocytic tumors, (**B**) ependymomas. One subependymoma was discovered to harbor 315 unique somatic mutations and was held out from this analysis. Unpaired t-test was used to test for significance between high-grade gliomas (HGG) and low-grade gliomas (LGG). ANOVA was used to test for significance between ependymoma subtypes. SNVs, single-nucleotide variants. GBM, glioblastoma. AA, anaplastic astrocytoma. LG2, Grade II glioma. PA, pilocytic astrocytoma. Classic, classic ependymoma. MPE, myxopapillary ependymoma. SE, subependymoma.

**Figure 2:**
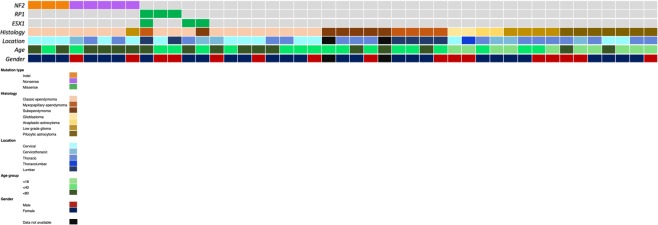
Heatmap depicting recurrent mutations and clinicopathologic correlates of IMSCT. NA, not available.

Tumors from 45 patients with IMSCTs were obtained and profiled with WES, observing an average of 12.6 SNVs per patient (SD, 46.3). Astrocytomas had an average of 5.4 SNVs (SD, 5.3) while ependymomas had an average of 16.5 SNVs (SD, 57.5; Fig. 1). All somatic mutations can be found in Supplementary Table 1. In this cohort of IMSCTs, patients were tumors were more common in females than males (female, N = 28; male, N = 15), mean age at surgery was 35.5 ± 19.6 years, and mean follow up was 17.4 months following surgery. We observed that 14 tumors were located in the cervical spine, six in the cervicothoracic spine, 16 in the thoracic spine, one in the thoracolumbar spine, and six in the lumbar spine. The mean number of spinal levels involved was 3.7 ± 2.2. Clinicopathologic characteristics are depicted in Fig. 2 and detailed in Supplementary Table 2.

### Ependymomas

The ependymoma WES cohort was comprised of classic ependymomas (N = 18), subependymomas (N = 6), and myxopapillary ependymomas (N = 5). The mean number of high-quality bases sequenced per sample was 1.13 × 10^11^, with an average of 118 distinct reads per targeted base. On average, 93.5% of these bases had at least 10 distinct reads. The average number of SNVs per patient did not vary significantly between ependymoma subtypes (Fig. 1B, *P* = 0.984). We observed recurrent indel and nonsense *NF1* mutations in classic ependymomas (38.9%). The other two recurrently mutated genes in our IMSCT cohort, *RP1* and *ESX1*, were observed in ependymomas as well. With missense *RP1* mutations observed in two classic ependymomas and one myxopapillary ependymoma and missense *ESX1* mutations observed in one classic ependymoma, one myxopapillary ependymoma, and one subependymoma (Fig. 2).

We took all somatic mutations (N = 108) across classic ependymomas but did not identify any significantly enriched pathways or molecular and cellular functions. (Supplementary Table 3). Similarly, pooling across 31 somatic mutations in myxopapillary ependymomas did not identify significantly enriched IPA results (Supplementary Table 3). However, across subependymomas, we pooled 24 total somatic mutations, and identified pathways involving Gs signaling, nNOS signaling, netrin signaling, and Methionine salvage. One subependymoma was discovered to harbor 315 unique somatic mutations and was thus separately analyzed. Top pathways in this tumor included caveolar-mediated endocytosis signaling and the neuroprotective role of THOP1 in Alzheimer’s disease. Cell-to-cell signaling, and interaction and cellular function and maintenance were the top molecular and cellular functions involved (Supplementary Table 3).

Recurrent copy number variations (CNVs) were identified only in classic ependymomas and myxopapillary ependymomas (Supplementary Table 4**)**. The majority of the recurrent CNVs were in classic ependymomas but none affecting known cancer driver genes. Of note, two (CGLI 13 and CGLI 25) of five myxopapillary ependymomas had five-fold or greater amplifications in *CTU1*, a gene involved in mRNA translation^23^.

### Astrocytomas

The astrocytoma cohort comprised of high-grade gliomas (glioblastoma, N = 2; anaplastic astrocytoma, N = 2), and low-grade gliomas (grade II astrocytoma, N = 5; pilocytic astrocytoma, N = 7;). Sequencing yielded 6.73 × 10^10^ high quality bases with 150 average distinct reads, of which on average 94% had at least 10 distinct reads. We found that high grade spinal gliomas (glioblastoma and anaplastic astrocytoma), compared to low grade spinal gliomas (WHO Grade II and pilocytic astrocytoma), had significantly more SNVs (Fig. 1A, *P* = 0.014). No recurrent mutations were identified in any astrocytoma subtype. Notably, no mutations in *BRAF* were noted in any astrocytoma sample.

We utilized 24 unique somatic mutations observed in spinal pilocytic astrocytomas for IPA, implicating a tRNA charging pathway and cell signaling molecular and cellular function (Supplementary Table 5). While no enriched pathways were identified among Grade II astrocytomas, among spinal anaplastic astrocytomas, we observed significant enrichment of cell death and survival, cellular growth and proliferation, cellular development, cellular function and maintenance, and cell cycle molecular and cellular functions (Supplementary Table 5)

### Whole genome sequencing of spinal ependymomas and astrocytomas

**Figure 3:**
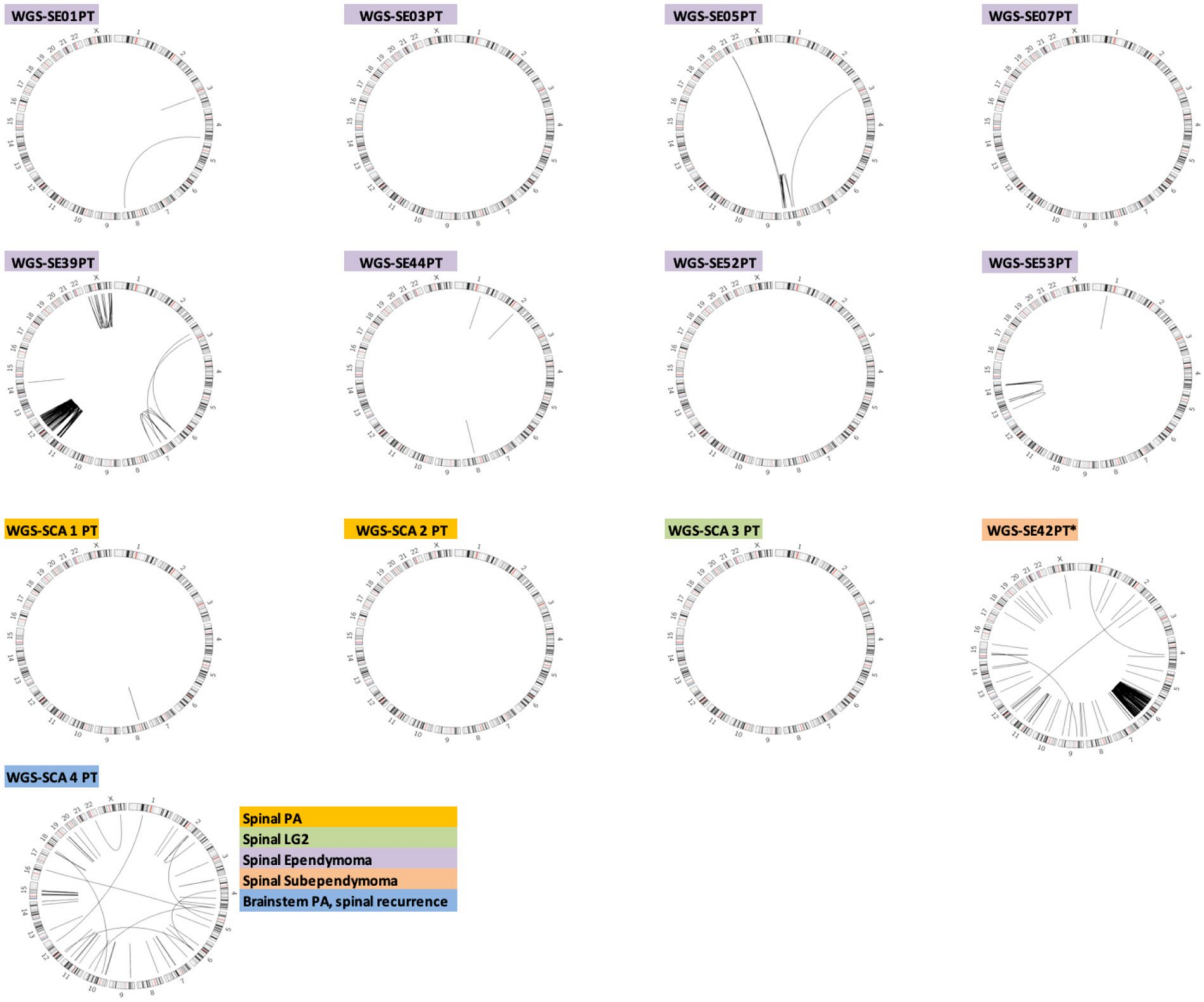
*Circos plots* depicting structural variants in 12 IMSCT selected for WGS and one brain stem glioma with recurrence in the cervical spine. PA, pilocytic astrocytoma. LG2, Grade II glioma.

In an effort to discover structural variants that may be integral to the oncogenesis of IMSCT, we performed whole genome sequencing of eight classic ependymomas, two pilocytic astrocytomas, one grade II low-grade glioma, and one subependymoma. Four of the ependymomas (SE01PT, SE03PT, SE05PT, SE07PT and SE09P) had undergone WES and have been previously described^14^. The mutations described in this manuscript are those discovered from WGS. WGS confirmed mutations identified via WES, identifying recurrent *NF2* mutations in spinal ependymoma. Structural variation was exceedingly rare in spinal pilocytic astrocytomas (Fig. 3). Structural variation among classic ependymoma ranged from entirely silent genomes to multiple intra- and interchromosomal rearrangements (Fig. 3). We further sought to characterize the genomic landscape of one patient (WGS-SCA 4 PT), who initially presented with brainstem pilocytic astrocytoma that recurred in the cervical spine. WGS was performed on the recurrent tumor, revealing mutations associated with intracranial pilocytic astrocytoma, including *BRAF V600E* and canonical *hTERT* promoter mutation.

## Discussion

In this study, we performed WES on 45 IMSCTs and WGS on 12 IMSCTs, representing the most comprehensive genetic description of IMSCTs to date. Our results demonstrate that IMSCTs of comparable histopathology to their intracranial counterparts have distinct mutational profiles and may have different genetic origins.

These findings are consistent with those of recent studies, which suggest that ependymomas arising from different compartments of the CNS display divergent molecular signatures. The majority of supratentorial ependymomas, for example, exhibit *C11orf95-RELA* fusion as a result of chromothripsis at chromosome 11q12.1-11q13.3^24,25^. In contrast, two distinct molecular subgroups of infratentorial ependymomas have emerged. Group A (GpA) tumors occur mainly in young, male patients and are more likely to be World Health Organization (WHO) grade III, with an overall decreased 5-year survival compared to Group B (GpB) tumors. GpA tumors feature a generally balanced genome, with occasional gain of 1q, while GpB tumors typically display numerous chromosomal aberrations. Further, overexpression of *LAMA* was found in GpA tumors, while GpB lesions demonstrated overexpression of *NELL2*^15,26,27^. Epigenetic studies also demonstrated that GpA tumors feature a CpG island methylator phenotype ultimately leading to trimethylation of H3K27, as well as epigenetic silencing of *LDOC1* with constitutive activation of NFkB and chronic Il-6 secretion^28,29^. More elaborate evaluation has led to further classification of ependymomas into 9 molecular subgroups according to age, CNS location, and biology, with important implications for prognostic stratification^30^.

In our study, the number of somatic mutations did not vary significantly across spinal ependymoma subtypes. Notably, we did not identify somatic mutations associated with intracranial ependymomas. The most commonly observed recurrent somatic mutation in spinal ependymomas were indel and nonsense *NF2* mutations in classic ependymomas (38.9%), consistent with several previous reports^6,31,32^. Two other genes, *RP1* and *ESX1*, were observed in spinal ependymomas (*RP1*, N = 2; *ESX1*, N = 3). In addition, two myxopapillary ependymomas demonstrate amplification of *CTU1*, a gene critically involved in modification at the wobble position of U34 of certain tRNAs. U34 enzymes have been shown to be upregulated in human colon and breast cancers. It is hypothesized that upregulation of U34 tRNA modifying enzymes allows for translation of proinvasive proteins in cancer cells^33^. In addition, recent work by Rapino and colleagues demonstrated that shRNA mediated knockdown of CTU1 in melanoma cells markedly decreased the viability of melanoma but not normal melanocytes^23^. Additionally, WGS of eight spinal ependymomas did not reveal evidence of *RELA* fusions, observed in supratentorial ependymoma and associated with poor prognosis^24^. observed in more than 60% of intracranial pilocytic astrocytomas These results demonstrate that spinal ependymomas of all subtypes display relatively few somatic mutations and emphasize the need for further investigation of epigenetic variations and copy number aberrations that may play a role in tumor pathogenesis^30^. While IPA did not identify enriched pathways associated with classic or myxopapillary ependymomas, we observed enrichment of Gs signaling, nNos signaling, Netrin signaling, and Methionine salvage II pathway in subependymomas.

Intracranial low-grade astrocytomas are characterized by mutations in *IDH1/2* and high-grade astrocytomas display numerous genetic alterations, including *EGFR*, *p53*, *hTERT*, *PTEN* and *CDKN2A/B*^34–36^. While several studies have evaluated the genetic basis of intracranial astrocytomas, none have focused exclusively on intramedullary astrocytomas given their rarity and difficulty obtaining sufficient pathologic samples for analysis. One prior report demonstrated mutations in *p16* (chromosome 9p21), *PTEN* (chromosome 10q23) and *BRAF* in spinal astrocytomas, although tumors were analyzed in combination with other midline PAs^37^.

Our analysis of 13 low-grade intramedullary astrocytomas yielded few somatic mutations and no recurrent genetic alterations. Interestingly, none of the LGGs demonstrated *IDH1/2* mutations. In 4 HGGs (2 WHO grade III, 2 WHO grade IV), there was one tumor featuring an *EGFR* mutation and one with a *p53* mutation. Recently, *BRAFV600E* mutations have been demonstrated in several intracranial tumors, including adult and pediatric PAs, grade II-IV pediatric astrocytomas, up to one-third of diencephalic PAs, as well as intracranial GGs^38,39^. The rare presence of *BRAF* mutations in spinal GGs has also been described^20^. In our cohort, canonical *BRAF* mutations were absent across astrocytoma samples and only one of four GG specimens featured the *BRAFV600E* mutation. Similarly, WGS of two spinal pilocytic astrocytomas did not reveal evidence of *KIAA1549-BRAF* fusions, which are observed in more than 60% of intracranial pilocytic astrocytomas^40^, but described less frequently in low grade spinal astrocytomas (25–40%)^41,42^.

No *H3F3A or HIST1H3B/HIST1H3C* mutations (encoding histone H3 variants, H3.3 and H3.1, respectively) were observed, counter to what has been previously reported for spinal glioblastoma^16,43,44^. We hypothesize this may be due to the small number of high-grade glioma cases (N = 4) analyzed in the current study or limited sensitivity of WES. Previous studies detected histone mutations in high grade spinal astrocytomas via immunohistochemistry^44,45^ or with targeted sequencing, which may enable greater sequencing depth and improved sensitivity^42^. To investigate this, we performed immunohistochemical staining for H3K27M on a subset of our cohort (pilocytic astrocytoma, N = 5; GBM, N = 2, anaplastic astrocytoma, N = 2). As anticipated 0/5 pilocytic astrocytomas stained positive for the mutation; however, 1/2 GBM and 1/2 anaplastic astrocytoma samples stained positive. We posit this may be due to inadequate genomic coverage over this section of the genome via WES, reinforcing the need for the higher depths than can be cost-effectively achieved with targeted sequencing panels. An alternative consideration, is that the sequencing depth we achieved is insufficient for identification of putative subclonal histone mutations, which have previously been described in adult midline gliomas^41^.

In spinal astrocytomas, the absence of genetic mutations critical to intracranial astrocytoma development and progression suggests that these intramedullary lesions are driven by alternative mechanisms of tumorigenesis. In fact, our results indicate that this divergence of molecular profiles is apparent in several IMSCT subtypes when compared with their intracranial counterparts. Considering the case of a patient with cervico-medullary pilocytic astrocytoma that recurred in the cervical spine (WGS-SCA 4 PT) reinforces this point. This case harbors the canonical intracranial pilocytic astrocytoma, even after recurrence in the spine, suggesting the origin was within the medulla and not the spinal cord. Our results suggest that genomic profiles, not classical anatomic considerations, may most clearly indicate a tumor’s origin and form the basis of targeted therapies.

An increasing body of evidence supports the notion that histologically indistinguishable tumors arising from different regions of the CNS display heterogeneous molecular signatures. The pathophysiology underlying this theory may be explained by the hypothesis that these tumors arise from progenitor cells unique to their tissue of origin, with resultant heterogeneity in their biological behavior. Taylor and colleagues in 2005 demonstrated that ependymomas in different areas (supratentorial, posterior fossa, spinal) expressed heterogeneous genetic profiles that corresponded to expression patterns of progenitor cells arising from those specific regions of the CNS. Their findings implicated radial glia cells, the precursor cells of normal ependymal cells, as the tumor-initiating cells of ependymomas arising from different areas of the CNS. They deduced that genetic upregulation of NOTCH and EphB cellular signaling pathways were a feature of supratentorial ependymomas, while aberrations in the HOX transcription factor family were identified in spinal ependymomas^18^. Other groups have also demonstrated heterogeneity in the molecular profiles of ependymomas, as well as other types of tumors from varying CNS compartments^46–49^.

Further analysis of epigenetic molecular profiles in human ependymomas of various locations has led to the proposed stratification of ependymomas into nine variants based on DNA methylation patterns, including supratentorial, posterior fossa, and spinal ependymomas, with three subgroups in each category^30^. These data suggest that molecular profiling of such tumors can help with their risk stratification, with significant implications for clinical prognostication and more focused routes of investigation. Our results strongly support the concept that histologically indistinguishable tumors from different parts of the CNS have unique molecular and genetic profiles and imply that robust genomic analysis may increase the diagnostic and prognostic accuracy for particular tumor subtypes.

This study is not without limitations. Despite being the largest study on IMSCT to date, the absolute numbers of cases for each tumor type was limited due to the rarity of these neoplasms. In addition, this study exclusively studied genetic alterations and did not focus on epigenetic or expression based changes, all of which play important roles in neoplasia. We anticipate that future studies will examine these potential areas of interest and that a more sophisticated understanding of the nonuniform molecular signatures associated with IMSCTs can allow for more precise modes of diagnostic and therapeutic intervention.

## Methods

### Sample acquisition

All samples were obtained after patients signed informed consent under a Johns Hopkins University School of Medicine Institutional Review Board (IRB)-approved study. All procedures were approved by the relevant ethics committees, and were performed in accordance with institutional guidelines and regulations. Fresh frozen or formalin-fixed, paraffin-embedded samples were obtained from patients following resection along with matching peripheral blood samples. Tumor pathological diagnoses were confirmed by a board-certified neuropathologist. Tumors were macro-dissected to ensure tumor content was >70%.

### Sample preparation and next-generation sequencing

Sample preparation, library construction, next generation sequencing, and bioinformatics analyses of tumor and normal samples were performed as previously described^14^. Briefly, DNA was extracted from frozen or formalin-fixed paraffin embedded (FFPE) tissue, along with matched blood samples using the Qiagen DNA FFPE tissue kit or Qiagen DNA blood mini kit (Qiagen, CA). Genomic DNA from tumor and normal samples were fragmented and used for Illumina TruSeq library construction (Illumina, San Diego, CA) according to the manufacturer’s instructions. Briefly, 50 nanograms (ng) - 3 micrograms (µg) of genomic DNA in 100 microliters (µl) of TE was fragmented in a Covaris sonicator (Covaris, Woburn, MA) to a size of 150–450 bp.

To remove fragments smaller than 150 bp, DNA was purified using Agencourt AMPure XP beads (Beckman Coulter, IN) in a ratio of 1.0 to 0.9 of PCR product to beads twice and washed using 70% ethanol per the manufacturer’s instructions. Purified, fragmented DNA was mixed with 36 µl of H2O, 10 µl of End Repair Reaction Buffer, 5 µl of End Repair Enzyme Mix (cat# E6050, NEB, Ipswich, MA). The 100 µl end-repair mixture was incubated at 20 °C for 30 min, and purified using Agencourt AMPure XP beads (Beckman Coulter, IN) in a ratio of 1.0 to 1.25 of PCR product to beads and washed using 70% ethanol per the manufacturer’s instructions. To A-tail, 42 µl of end-repaired DNA was mixed with 5 µl of 10X dA Tailing Reaction Buffer and 3 µl of Klenow (exo-)(cat# E6053, NEB, Ipswich, MA). The 50 µl mixture was incubated at 37 °C for 30 min and purified using Agencourt AMPure XP beads (Beckman Coulter, IN) in a ratio of 1.0 to 1.0 of PCR product to beads and washed using 70% ethanol per the manufacturer’s instructions. For adaptor ligation, 25 µl of A-tailed DNA was mixed with 6.7 µl of H2O, 3.3 µl of PE-adaptor (Illumina), 10 µl of 5X Ligation buffer and 5 µl of Quick T4 DNA ligase (cat# E6056, NEB, Ipswich, MA). The ligation mixture was incubated at 20 °C for 15 min and purified using Agencourt AMPure XP beads (Beckman Coulter, IN) in a ratio of 1.0 to 0.95 and 1.0 of PCR product to beads twice and washed using 70% ethanol per the manufacturer’s instructions.

To obtain an amplified library, twelve PCRs of 25 µl each were set up, each including 15.5 µl of H2O, 5 µl of 5 x Phusion HF buffer, 0.5 µl of a dNTP mix containing 10 mM of each dNTP, 1.25 µl of DMSO, 0.25 µl of Illumina PE primer #1, 0.25 µl of Illumina PE primer #2, 0.25 µl of Hotstart Phusion polymerase, and 2 µl of the DNA. The PCR program used was: 98 °C for 2 minutes; 12 cycles of 98 °C for 15 seconds, 65 °C for 30 seconds, 72 °C for 30 seconds; and 72 °C for 5 min. DNA was purified using Agencourt AMPure XP beads (Beckman Coulter, IN) in a ratio of 1.0 to 1.0 of PCR product to beads and washed using 70% ethanol per the manufacturer’s instructions. Exonic regions were captured in solution using the Agilent SureSelect v.4 kit according to the manufacturer’s instructions (Agilent, Santa Clara, CA). The captured library was then purified with a Qiagen MinElute column purification kit and eluted in 17 µl of 70 °C EB to obtain 15 µl of captured DNA library. The captured DNA library was amplified in the following way: Eight 30uL PCR reactions each containing 19 µl of H2O, 6 µl of 5 x Phusion HF buffer, 0.6 µl of 10 mM dNTP, 1.5 µl of DMSO, 0.30 µl of Illumina PE primer #1, 0.30 µl of Illumina PE primer #2, 0.30 µl of Hotstart Phusion polymerase, and 2 µl of captured exome library were set up. The PCR program used was: 98 °C for 30 seconds; 14 cycles (exome) of 98 °C for 10 seconds, 65 °C for 30 seconds, 72 °C for 30 seconds; and 72 °C for 5 min. To purify PCR products, a NucleoSpin Extract II purification kit (Macherey-Nagel, PA) was used following the manufacturer’s instructions. Paired-end sequencing, resulting in 100 bases from each end of the fragments was performed using Illumina HiSeq. 2500 and instrumentation (Illumina, San Diego, CA).

### Processing of next-generation sequencing data and identification of putative somatic mutations

Somatic mutations were identified using VariantDx custom software for identifying mutations in matched tumor and normal samples. Prior to mutation calling, primary processing of sequence data for both tumor and normal samples were performed using Illumina CASAVA software (v1.8), including masking of adapter sequences. Sequence reads were aligned against the human reference genome (version hg18) using ELAND software. Candidate somatic mutations, consisting of point mutations, insertions, and deletions were then identified using VariantDx. VariantDx examines sequence alignments of tumor samples against a matched normal while applying filters to exclude alignment and sequencing artifacts. In brief, an alignment filter was applied to exclude quality failed reads, unpaired reads, and poorly mapped reads in the tumor. A base quality filter was applied to limit inclusion of bases with reported phred quality score >30 for the tumor and >20 for the normal. A mutation in the tumor was identified as a candidate somatic mutation only when (i) distinct paired reads contained the mutation in the tumor; (ii) the number of distinct paired reads containing a particular mutation in the tumor was at least 10% of the total distinct read pairs (iii) the mismatched base was not present in >1% of the reads in the matched normal sample as well as not present in a custom database of common germline variants derived from dbSNP and (iv) the position was covered in both the tumor and normal. Mutations arising from misplaced genome alignments, including paralogous sequences, were identified and excluded by searching the reference genome.

Candidate somatic mutations were further filtered based on gene annotation to identify those occurring in protein coding regions. Functional consequences were predicted using snpEff and a custom database of CCDS, RefSeq and Ensembl annotations using the latest transcript versions available on hg18 from UCSC (https://genome.ucsc.edu/). Predictions were ordered to prefer transcripts with canonical start and stop codons and CCDS or Refseq transcripts over Ensembl when available. Finally, mutations were filtered to exclude intronic and silent changes, while retaining mutations resulting in missense mutations, nonsense mutations, frameshifts, or splice site alterations. A manual visual inspection step was used to further remove artifactual changes.

### Copy number alteration identification

Copy number alterations were identified by comparing normalized average per-base distinct (duplicates removed) coverage for a particular gene in a DNA sample to the normalized average per-base distinct coverage for the gene in the patient-matched normal DNA sample.

### Structural variant identification

Structural analyses of genomic rearrangements were performed as previously described^50^. Briefly, somatic rearrangements were identified by querying aberrantly mapping paired-end reads. The discordantly mapping pairs were grouped into 1 kb bins when at least 2 distinct tag pairs (with distinct start sites) spanned the same two 1 kb bins (known bins which contained aberrantly mapping tags were removed, as well as 1 kb bins involved in known germline structural alterations). To identify all high-confidence genomic rearrangements, candidate rearrangements were filtered using the above described criteria and were required to harbor at least 7 distinct tag pairs spanning the candidate breakpoint, as well as a distribution of mapping positions on either side of the candidate breakpoint (>50 bp). Breakpoints were further characterized, where possible, using BLAT alignment to the human genome sequence.

### Pathway analysis

Bioinformatic pathway analysis was performed on the set of mutations identified for each individual tumor using the Ingenuity Pathway Analysis and Knowledge Base (QIAGEN Bioinformatics, Redwood City, CA). With this tool, distinct canonical pathways and upstream regulators were identified in an attempt to elucidate possible dysregulated cellular pathways as a result of mutations within tumor subtypes. Pathways were considered significantly enriched only for after correction for multiple hypothesis testing.

## Supplementary information

Supplementary Table 1

Supplementary Table 2

Supplementary Table 3

Supplementary Table 4

Supplementary Table 5
