# Supplementary material for: Genomic Landscape of Intramedullary Spinal Cord Gliomas
Source: Sci Rep. 2019 Dec 10;9:18722. doi: 10.1038/s41598-019-54286-9 (PMC6904446; doi:10.1038/s41598-019-54286-9)
Supplement: Supplementary file 1 — Supplementary Table 1 [file 41598_2019_54286_MOESM1_ESM.pdf]

Genomic Landscape of Intramedullary Spinal Cord Gliomas

Ming Zhang, Ph.D.[1,+], Rajiv R. Iyer, M.D.[2,+], Tej D. Azad M.D., M.S. [2,3,+], Qing Wang, Ph.D.[1], Tomas Garzon-Muvdi, M.D.[2,4], Joanna Wang M.D.[5], Ann Liu M.D.[2], Peter Burger M.D.[6], Charles Eberhart M.D.,PhD[6], Fausto J. Rodriguez[6], M.D., Daniel M. Sciubba M.D.[2], Jean-Paul Wolinsky M.D.[2,7] , Ziya Gokaslan M.D.[2,8], Mari Groves M.D.[2], George I. Jallo, M.D. [2,9,*], Chetan Bettegowda, M.D., Ph.D.[1,2*]

Supplementary Table 1A Somatic Mutations in Astrocytomas

Tumor	Sample ID	Gene	Gene Description	Gene Accession	Nucleotide	Amino Acid	Mutation Type
Pilocytic astrocytoma	WGS-SCA 1 PT	ABCA8	ATP-binding cassette, sub-family A (ABC1), member 8	CCDS11680.1	chr17 66878094-66878094 G A	1246R>C	Substitution
Anaplastic astrocytoma	7929T	ACTL9	actin-like 9	CCDS12207.1	chr19 8669058-8669058 C T	332A>T	Substitution
Glioblastoma	7960T	ADAMDEC1	ADAM-like, decysin 1	CCDS6044.1	chr8_24310850-24310850_G_T	188G>V	Substitution
Pilocytic astrocytoma	9922T	ADAMTS20	ADAM metallopeptidase with thrombospondin type 1 motif, 20	CCDS31778.2	chr12_43847717-43847717_G_A	585R>C	Substitution
Grade II astrocytoma	CGLI58PT1	ADCK2	aarF domain containing kinase 2	CCDS5861.1	chr7 140374534-140374534 T A	353F>I	Substitution
Pilocytic astrocytoma	CGLI44PT1	ALOX15	arachidonate 15-lipoxygenase	CCDS11049.1	chr17 4541604-4541604 C T	239V>M	Substitution
Anaplastic astrocytoma	8068T	C11orf88	chromosome 11 open reading frame 88	CCDS41712.1	chr11_110890860-110890860__T	140_141insT	Insertion
Pilocytic astrocytoma	7937T	C17orf98	chromosome 17 open reading frame 98	CCDS42310.1	chr17 36991477-36991477 C T	144V>I	Substitution
Grade II astrocytoma	7982T	CATSPERD	catsper channel auxiliary subunit delta	CCDS12149.2	chr19_5729644-5729644_C_T	785P>L	Substitution
Pilocytic astrocytoma	7937T	CCDC141	coiled-coil domain containing 141	NM 173648	chr2 179718299-179718299 T C	1038Y>C	Substitution
Pilocytic astrocytoma	SCA 2 PT	CCDC40	coiled-coil domain containing 40	ENST00000374877	chr17_78064092-78064092_G_A	996R>H	Substitution
Grade II astrocytoma	CGLI58PT1	CD177P1	CD177 molecule pseudogene 1	ENST00000378007	chr19_43882895-43882895_A_C	58V>G	Substitution
Grade II astrocytoma	CGLI58PT1	CDH11	cadherin 11, type 2, OB-cadherin (osteoblast)	ENST00000394156	chr16_64982664-64982664_A_C	641S>A	Substitution
Glioblastoma	CGLI 36	CDK17	cyclin-dependent kinase 17	CCDS9061.1	chr12 95241922-95241922 G A	73P>L	Substitution
Pilocytic astrocytoma	7937T	CDRT1	CMT1A duplicated region transcript 1	NM 006382	chr17 15457962-15457962 G C	261L>V	Substitution
Glioblastoma	7960T	CFTR	cystic fibrosis transmembrane conductance regulator (ATP-binding cassette sub-family C, member 7)	CCDS5773.1	chr7_116986919-116986919_G_A	520V>I	Substitution
Grade II astrocytoma	CGLI 39	CKAP2	cytoskeleton associated protein 2	CCDS41893.1	chr13 51933923-51933923 G A	322V>I	Substitution
Glioblastoma	CGLI 36	COL2A1	collagen; type II; alpha 1	CCDS41778.1	chr12 46663178-46663178 C T	687G>S	Substitution
Glioblastoma	CGLI 36	COL3A1	collagen; type III; alpha 1	CCDS2297.1	chr2 189558634-189558634 A	NA	Deletion
Glioblastoma	CGLI 36	CYP4A11	cytochrome P450; family 4; subfamily A; polypeptide 11	CCDS543.1	chr1 47171080-47171080 C T	435R>H	Substitution
Grade II astrocytoma	SCA 3 PT	DTX2	deltex homolog 2 (Drosophila)	CCDS5587.1	chr7 76109831-76109831 C T	2A>V	Substitution
Anaplastic astrocytoma	7929T	EGFR	epidermal growth factor receptor	CCDS5514.1	chr7 55233903-55233903 G A	NA	Substitution
Anaplastic astrocytoma	7929T	EMR3	egf-like module containing, mucin-like, hormone receptor-like 3	CCDS12315.1	chr19_14626978-14626978_C_T	NA	Substitution
Anaplastic astrocytoma	7929T	EXT1	exostosin glycosyltransferase 1	CCDS6324.1	chr8 119192067-119192067 T C	134I>V	Substitution
Glioblastoma	7960T	FABP7	fatty acid binding protein 7, brain	CCDS5127.1	chr6 123142702-123142702 A C	22K>Q	Substitution
Anaplastic astrocytoma	7929T	FAM171A1	family with sequence similarity 171, member A1	CCDS31154.1	chr10_15295567-15295567_C_T	676A>T	Substitution
Anaplastic astrocytoma	Ex-7929T	FLG2	filaggrin family member 2	CCDS30861.1	chr1 150595613-150595613 G T	425Q>K	Substitution
Grade II astrocytoma	7982T	FOXP3	forkhead box P3	CCDS14323.1	chrX 48996560-48996560 G T	339P>T	Substitution
Glioblastoma	7960T	GOLGA3	golgin A3	CCDS9281.1	chr12 131885140-131885140 A	NA	Deletion
Anaplastic astrocytoma	7929T	GPATCH2	G patch domain containing 2	CCDS1518.1	chr1 215860046-215860046 G A	159R>C	Substitution
Pilocytic astrocytoma	SCA 2 PT	GRB7	growth factor receptor-bound protein 7	CCDS11345.1	chr17 37899484-37899484 C T	172P>L	Substitution
Pilocytic astrocytoma	SCA 2 PT	HIPK1	homeodomain interacting protein kinase 1	CCDS867.1	chr1 114495432-114495432 A G	374D>G	Substitution
Pilocytic astrocytoma	SCA 2 PT	HMCN2	hemicentin 2	ENST00000277491	chr9_133271664-133271664_G_A	622R>Q	Substitution
Grade II astrocytoma	CGLI58PT1	HPS6	Hermansky-Pudlak syndrome 6	CCDS7527.1	chr10_103826243-103826243_G_A	338G>S	Substitution
Pilocytic astrocytoma	SCA 2 PT	IARS2	isoleucyl-tRNA synthetase 2, mitochondrial	CCDS1523.1	chr1 220300143-220300143 A G	599I>V	Substitution
Anaplastic astrocytoma	7929T	ITGA1	integrin, alpha 1	CCDS3955.1	chr5 52254470-52254470 A C	800E>A	Substitution
Pilocytic astrocytoma	CGLI44PT1	ITGA11	integrin, alpha 11	CCDS45291.1	chr15 68619063-68619063 G A	714R>X	Substitution
Glioblastoma	CGLI 36	KCNH7	potassium voltage-gated channel; subfamily H (eag-related); member 7	CCDS2219.1	chr2_162965109-162965109_C_T	748G>E	Substitution
Glioblastoma	7960T	KIAA0556	KIAA0556	CCDS32415.1	chr16 27622776-27622776 G A	449G>S	Substitution
Anaplastic astrocytoma	7929T	LENG9	leukocyte receptor cluster (LRC) member 9	CCDS12895.1	chr19 59665989-59665989 G A	178A>V	Substitution
Grade II astrocytoma	CGLI58PT1	LGI2	leucine-rich repeat LGI family, member 2	CCDS3431.1	chr4 25032262-25032264 CAG	NA	Deletion
Grade II astrocytoma	CGLI 02	LRP1	Prolow-density lipoprotein receptor-related protein 1 precursor (LRP) (Alpha-2-macroglobulin receptor) (A2MR) (Apolipoprotein E receptor) (APOER) (CD91 antigen) [Contains: Low-density lipoprotein receptor- related protein 1 85 kDa subunit (LRP-85); Low-de	CCDS8932.1	chr12_55884716-55884716_A_C	3704K>T	Substitution
Grade II astrocytoma	CGLI 02	LRP1	Prolow-density lipoprotein receptor-related protein 1 precursor (LRP) (Alpha-2-macroglobulin receptor) (A2MR) (Apolipoprotein E receptor) (APOER) (CD91 antigen) [Contains: Low-density lipoprotein receptor- related protein 1 85 kDa subunit (LRP-85); Low-de	CCDS8932.1	chr12_55884728-55884728_T_G	3708V>G	Substitution
Pilocytic astrocytoma	CGLI 15	MARS	methionyl-tRNA synthetase	CCDS8942.1	chr12 56191866-56191866 C T	496T>I	Substitution
Anaplastic astrocytoma	7929T	MMP20	matrix metallopeptidase 20	CCDS8318.1	chr11_101982562-101982562_C_G	289K>N	Substitution
Grade II astrocytoma	CGLI58PT1	MSRB3	methionine sulfoxide reductase B3	CCDS8973.1	chr12 65847577-65847577 T C	128M>T	Substitution
Anaplastic astrocytoma	7929T	MXRA5	matrix-remodelling associated 5	CCDS14124.1	chrX 3245528-3245528 A T	2065I>N	Substitution
Glioblastoma	CGLI 36	MYOM1	myomesin-1 isoform a	NM 003803	chr18 3205068-3205068 C T	52A>T	Substitution
Glioblastoma	CGLI 36	MYT1	myelin transcription factor 1	CCDS13558.1	chr20 62324910-62324910 G T	800C>F	Substitution
Anaplastic astrocytoma	7929T	NARG2	NMDA receptor regulated 2	NM 001276385	chr15 58533074-58533074 A	NA	Deletion
Grade II astrocytoma	CGLI 39	NF2	neurofibromin 2 (merlin)	CCDS13861.1	chr22 28381658-28381658 C T	198R>X	Substitution
Pilocytic astrocytoma	7937T	NOL4	nucleolar protein 4	CCDS11907.2	chr18 31537346-31537346 G C	458R>G	Substitution
Anaplastic astrocytoma	8068T	NOP58	NOP58 ribonucleoprotein	CCDS2353.1	chr2 202855420-202855420 A G	93K>E	Substitution
Pilocytic astrocytoma	7739T	NOTCH2NL	notch 2 N-terminal like	CCDS909.1	chr1_143992990-143992990_C_A	188P>H	Substitution
Grade II astrocytoma	7982T	NYNRIN	NYN domain and retroviral integrase containing	NM 025081	chr14 23953797-23953797 A T	1001D>V	Substitution
Pilocytic astrocytoma	9922T	OGFR	opioid growth factor receptor	CCDS13504.1	chr20 61444637-61444637 G C	557S>T	Substitution
Anaplastic astrocytoma	7929T	OLIG2	oligodendrocyte lineage transcription factor 2	CCDS13620.1	chr21 33321908-33321908 G	NA	Insertion
Pilocytic astrocytoma	SCA 1 PT	OR10H1	olfactory receptor, family 10, subfamily H, member 1	CCDS12335.1	chr19 15918238-15918238 A C	204C>G	Substitution
Anaplastic astrocytoma	7929T	OR4D5	olfactory receptor, family 4, subfamily D, member 5	CCDS31699.1	chr11_123316302-123316302_G_A	257V>I	Substitution
Pilocytic astrocytoma	7937T	OR9Q1	olfactory receptor, family 9, subfamily Q, member 1	CCDS31543.1	chr11 57947443-57947443 T A	176F>Y	Substitution
Pilocytic astrocytoma	7739T	PANK3	pantothenate kinase 3	CCDS4368.1	chr5 167921011-167921011 T A	301I>F	Substitution
Grade II astrocytoma	CGLI 39	PDLIM4	PDZ and LIM domain 4	CCDS4152.1	chr5 131635715-131635715 T A	296L>Q	Substitution
Glioblastoma	7960T	PER3	period circadian clock 3	CCDS89.1	chr1_7802762-7802762_A_T	545R>S	Substitution
Anaplastic astrocytoma	7929T	PGAP1	post-GPI attachment to proteins 1	CCDS2318.1	chr2 197489418-197489418 T G	149E>A	Substitution
Grade II astrocytoma	CGLI 02	PIGM	phosphatidylinositol glycan anchor biosynthesis; class M	CCDS1192.1	chr1 158267172-158267172 T A	328N>Y	Substitution
Grade II astrocytoma	CGLI 02	PIGM	phosphatidylinositol glycan anchor biosynthesis; class M	CCDS1192.1	chr1 158267173-158267173 A C	327F>L	Substitution
Grade II astrocytoma	CGLI 02	PLA2G4D	phospholipase A2; group IVD (cytosolic)	CCDS32203.1	chr15 40165767-40165767 A C	108L>R	Substitution
Pilocytic astrocytoma	9922T	POLR2A	polymerase (RNA) II (DNA directed) polypeptide A, 220kDa	CCDS32548.1	chr17 7400213-7400213 A C	223E>A	Substitution
Anaplastic astrocytoma	7929T	PPM1D	protein phosphatase, Mg2+/Mn2+ dependent, 1D	CCDS11625.1	chr17 56095291-56095291 G T	472E>X	Substitution
Glioblastoma	CGLI 36	PRKCA	protein kinase C; alpha	CCDS11664.1	chr17 62215443-62215443 C T	580R>W	Substitution
Pilocytic astrocytoma	CGLI44PT1	RASSF2	Ras association (RalGDS/AF-6) domain family member 2	CCDS13083.1	chr20 4771131-4771131 C T	168R>H	Substitution
Anaplastic astrocytoma	7929T	RWDD4	RWD domain containing 4	CCDS34111.1	chr4 184807718-184807718 A	NA	Deletion
Anaplastic astrocytoma	7929T	SLCO4A1	solute carrier organic anion transporter family, member 4A1	CCDS13501.1	chr20 60758459-60758459 C T	70R>W	Substitution

Pilocytic astrocytoma	7937T	TBX2	T-box 2	CCDS11627.2	chr17_59481804-59481804_A_T	278N>I	Substitution
Grade II astrocytoma	7982T	TECTA	tectorin alpha	CCDS8434.1	chr11_120505572-120505572_G_A	795V>M	Substitution
Anaplastic astrocytoma	7929T	TMEM128	transmembrane protein 128	CCDS3373.1	chr4 4298906-4298906 A G	31W>R	Substitution
Glioblastoma	CGLI 36	TP53	tumor protein p53	CCDS11118.1	chr17_7518264-7518264_G_A	248R>W	Substitution
Pilocytic astrocytoma	7937T	TRIM32	tripartite motif containing 32	CCDS6817.1	chr9 119461139-119461139 T G	373L>R	Substitution
Pilocytic astrocytoma	9922T	UGT2B17	UDP glucuronosyltransferase 2 family, polypeptide B17	CCDS3523.1	chr4_69433908-69433908_A_C	99Y>D	Substitution
Anaplastic astrocytoma	7929T	UPP1	uridine phosphorylase 1	CCDS5507.1	chr7_48107974-48107974_G_A	64R>Q	Substitution
Anaplastic astrocytoma	7929T	USP1	ubiquitin specific peptidase 1	CCDS621.1	chr1_62685773-62685773_A_C	NA	Substitution
Glioblastoma	CGLI 36	VAX1	ventral anterior homeobox 1	CCDS7597.1	chr10_118881747-118881747_C_T	175R>H	Substitution
Anaplastic astrocytoma	7929T	VPS8	vacuolar protein sorting 8 homolog (S. cerevisiae)	NM 001009921	chr3 186224830-186224830 C G	1339P>A	Substitution
Anaplastic astrocytoma	8068T	WDYHV1	WDYHV motif containing 1	CCDS6344.1	chr8 124522813-124522813 C T	199R>W	Substitution
Glioblastoma	CGLI 36	WNT4	wingless-type MMTV integration site family; member 4	CCDS223.1	chr1 22319578-22319578 C T	203R>Q	Substitution
Anaplastic astrocytoma	7929T	ZEB2	zinc finger E-box binding homeobox 2	CCDS2186.1	chr2 144864029-144864029 G A	1035A>V	Substitution
Grade II astrocytoma	CGLI 02	ZNF513	zinc finger protein 513	CCDS1751.1	chr2 27455224-27455224 G A	138P>L	Substitution

Supplementary Table 1B. Somatic Mutations in Ependymomas

Tumor	Sample ID	Gene	Gene Description	Gene Accession	Nucleotide	Amino Acid	Mutation Type
Ependymoma	8648T	OASL	2'-5'-oligoadenylate synthetase-like	CCDS9211.1	chr12_121465422-121465422_C_T	286A>T	Substitution
Ependymoma	8648T	ATF1	activating transcription factor 1	CCDS8803.1	chr12 51189692-51189692 G A	32V>I	Substitution
Ependymoma	8129T	ADAM7	ADAM metallopeptidase domain 7	CCDS6045.1	chr8 24350689-24350689 G A	597D>N	Substitution
Ependymoma	SE01PT	ADAMTSL3	ADAMTS-like 3	CCDS10326.1	chr15_84553907-84553907_A_T	339I>F	Substitution
Ependymoma	8527T	AK5	adenylate kinase 5	CCDS675.1	chr1 77984336-77984336 G A	412R>H	Substitution
Ependymoma	8540T	ALKBH1	alkB, alkylation repair homolog 1 (E. coli)	CCDS32127.1	chr14 78170751-78170751 C T	85V>I	Substitution
Ependymoma	CGLI 11	AMDHD1	amidohydrolase domain containing 1	CCDS9057.1	chr12 94880327-94880327 G A	293V>I	Substitution
Ependymoma	SE01PT	ANK2	ankyrin 2, neuronal	CCDS3702.1	chr4_114257844-114257844_A_G	1235M>V	Substitution
Ependymoma	CGLI 11	ANKS3	ankyrin repeat and sterile alpha motif domain containing 3	CCDS10520.1	chr16_4688051-4688051_C_T	580E>K	Substitution
Ependymoma	SE39PT	ANKRD30B	ankyrin repeat domain 30B	NM 001145029	chr18 14754923-14754923 C A	179A>D	Substitution
Ependymoma	SE03PT	BLOC1S3	biogenesis of lysosomal organelles complex-1, subunit 3	CCDS12656.1	chr19_45682853-45682853_C_T	100P>L	Substitution
Ependymoma	8129T	BLOC1S4	biogenesis of lysosomal organelles complex-1, subunit 4, cappuccino	CCDS3393.1	chr4_6718111-6718111_C_T	59P>S	Substitution
Ependymoma	SE07PT	CPAMD8	C3 and PZP-like, alpha-2-macroglobulin domain containing 8	CCDS42519.1	chr19_17088318-17088318_C_T	587V>M	Substitution
Ependymoma	SE53PT	CEP85	centrosomal protein 85kDa	CCDS277.1	chr1_26582256-26582256_A_G	268Q>R	Substitution
Ependymoma	8648T	CHRNA10	cholinergic receptor, nicotinic, alpha 10 (neuronal)	CCDS7745.1	chr11_3692509-3692509_G_A	12L>F	Substitution
Ependymoma	7692T	C11orf80	chromosome 11 open reading frame 80	CCDS53664.1	chr11 66512291-66512291 GGC	26G>GA	Insertion
Ependymoma	8129T	C12orf4	chromosome 12 open reading frame 4	CCDS8528.1	chr12 4627288-4627288 T	S324Lfs*8	Deletion
Ependymoma	SE03PT	C19orf60	chromosome 19 open reading frame 60	CCDS42524.1	chr19 18699854-18699854 C T	50A>V	Substitution
Ependymoma	CGLI 11	COL11A2	Collagen alpha-2(XI) chain precursor.	CCDS43452.1	chr6 33255557-33255557 G A	369R>W	Substitution
Ependymoma	CGLI42PT1	COL6A1	collagen, type VI, alpha 1	CCDS13727.1	chr21_47417353-47417353_G_A	473G>R	Substitution
Ependymoma	CGLI 11	C5	complement component 5	CCDS6826.1	chr9 122799859-122799859 C T	859M>I	Substitution
Ependymoma	SE03PT	C6	complement component 6	CCDS3936.1	chr5 41153948-41153948 T G	752T>P	Substitution
Ependymoma	7924T	DGAT2	diacylglycerol O-acyltransferase 2	CCDS31642.1	chr11 75501254-75501254 T G	91L>R	Substitution
Ependymoma	8520T	DLG3	discs, large homolog 3 (Drosophila)	CCDS14403.1	chrX 69719065-69719065 A T	637K>M	Substitution
Ependymoma	SE44PT	DCDC5	doublecortin domain containing 5	ENST00000444572	chr11 30900261-30900261 G	NA	Deletion
Ependymoma	SE53PT	DNAH5	dynein, axonemal, heavy chain 5	CCDS3882.1	chr5 13753358-13753361 CTTT	NA	Deletion
Ependymoma	8129T	DMD	dystrophin	CCDS14233.1	chrX 33229397-33229397 A C	31+2T>G	Substitution
Ependymoma	8648T	EPB41L5	erythrocyte membrane protein band 4.1 like 5	CCDS2130.1	chr2_120836144-120836144_G_A	264G>S	Substitution
Ependymoma	CGLI 11	ESX1	ESX homeobox 1	CCDS14516.1	chrX 103381827-103381827 G C	320P>R	Substitution
Ependymoma	SE39PT	ETV3L	ets variant 3-like	NM 001004341	chr1 157062857-157062857 C T	224G>S	Substitution
Ependymoma	SE01PT	FAM118A	family with sequence similarity 118, member A	ENST00000405673	chr22_45724288-45724288_G_A	180A>T	Substitution
Ependymoma	SE44PT	FAM171A1	family with sequence similarity 171, member A1	CCDS31154.1	chr10_15290742-15290742_G_A	217P>L	Substitution
Ependymoma	8648T	FAM216A	family with sequence similarity 216, member A	CCDS31899.1	chr12_110924194-110924194_A_C	124N>T	Substitution
Ependymoma	SE44PT	FSIP2	fibrous sheath interacting protein 2	ENST00000424728	chr2_186662005-186662005_T_G	3381L>W	Substitution
Ependymoma	SE53PT	FOXO3	forkhead box O3	CCDS5068.1	chr6 108985481-108985481 G A	482S>N	Substitution
Ependymoma	7692T	FMNL1	formin-like 1	CCDS11497.1	chr17 43313517-43313517 A G	210H>R	Substitution
Ependymoma	SE52PT	FRK	fyn-related kinase	CCDS5103.1	chr6 116325151-116325151 C T	119G>R	Substitution
Ependymoma	CGLI 11	FGD2	FYVE; RhoGEF and PH domain containing 2	CCDS4829.1	chr6_37101585-37101585_A_C	500K>Q	Substitution
Ependymoma	CGLI42PT1	GPR137C	G protein-coupled receptor 137C	CCDS45106.1	chr14 53019944-53019944 A G	27S>G	Substitution
Ependymoma	CGLI42PT1	GPR139	G protein-coupled receptor 139	CCDS32398.1	chr16 20043965-20043965 A	NA	Deletion
Ependymoma	8520T	GRID1	glutamate receptor, ionotropic, delta 1	CCDS31236.1	chr10_87615782-87615782_G_A	1113+4C>T	Substitution
Ependymoma	8129T	GAREM	GRB2 associated, regulator of MAPK1	CCDS56057.1	chr18 29847854-29847854 C T	871G>S	Substitution
Ependymoma	8527T	HELZ2	helicase with zinc finger 2, transcriptional coactivator	CCDS33508.1	chr20_62195798-62195798_C_A	1459E>D	Substitution
Ependymoma	SE39PT	HIVEP3	human immunodeficiency virus type I enhancer binding protein 3	CCDS463.1	chr1_41984103-41984103_C_T	1814G>R	Substitution
Ependymoma	8129T	IGDCC4	immunoglobulin superfamily, DCC subclass, member 4	CCDS10206.1	chr15_65680819-65680819_T_C	938Q>R	Substitution
Ependymoma	SE52PT	INA	internexin neuronal intermediate filament protein, alpha	CCDS7545.1	chr10_105037278-105037278_C_G	104R>G	Substitution
Ependymoma	8648T	KRTAP17-1	keratin associated protein 17-1	CCDS11387.1	chr17_39471876-39471876_G_T	9F>L	Substitution
Ependymoma	7692T	KNDC1	kinase non-catalytic C-lobe domain (KIND) containing 1	CCDS7674.1	chr10_134996942-134996942_C_T	152A>V	Substitution
Ependymoma	SE44PT	LRRC8B	leucine rich repeat containing 8 family, member B	CCDS724.1	chr1_90058573-90058573_C_T	795R>C	Substitution
Ependymoma	8540T	METRN	meteorin, glial cell differentiation regulator	CCDS10422.1	chr16_765838-765838_G_T	120R>L	Substitution
Ependymoma	8648T	MYO10	myosin X	CCDS54834.1	chr5 16818265-16818265 G C	44Y>X	Substitution
Ependymoma	SE03PT	NAGS	N-acetylglutamate synthase	CCDS11473.1	chr17 42082165-42082165 C T	45P>L	Substitution
Ependymoma	8129T	NF2	neurofibromin 2 (merlin)	CCDS13861.1	chr22_30051648-30051648__GCACT	R196Cfs*15	Insertion
Ependymoma	8540T	NF2	neurofibromin 2 (merlin)	CCDS13861.1	chr22 30054212-30054212 C T	212Q>X	Substitution
Ependymoma	SE01PT	NF2	neurofibromin 2 (merlin)	CCDS13861.1	chr22_30051622-30051622_G_T	186E>X	Substitution
Ependymoma	SE07PT	NF2	neurofibromin 2 (merlin)	CCDS13861.1	chr22 30070841-30070841 C	NA	Deletion
Ependymoma	SE39PT	NF2	neurofibromin 2 (merlin)	CCDS13861.1	chr22_30067824-30067824_C_T	337Q>X	Substitution
Ependymoma	SE52PT	NF2	neurofibromin 2 (merlin)	CCDS13861.1	chr22_30051651-30051651_C_	NA	Deletion
Ependymoma	SE53PT	NF2	neurofibromin 2 (merlin)	CCDS13861.1	chr22 30067836-30067836 C T	341R>X	Substitution
Ependymoma	SE52PT	NEK8	NIMA-related kinase 8	CCDS32597.1	chr17 27064454-27064454 G C	250S>T	Substitution
Ependymoma	8129T	NINL	ninein-like	CCDS33452.1	chr20 25478852-25478852 T C	388Y>C	Substitution
Ependymoma	SE05PT	NLRP5	NLR family, pyrin domain containing 5	CCDS12938.1	chr19 56538351-56538351 G A	251R>H	Substitution
Ependymoma	SE53PT	NIPA1	non imprinted in Prader-Willi/Angelman syndrome 1	CCDS10011.1	chr15_23086365-23086367_GCC_	NA	Deletion
Ependymoma	8527T	NSUN6	NOP2/Sun domain family, member 6	CCDS7130.1	chr10 18885233-18885233 A C	227S>R	Substitution
Ependymoma	SE39PT	OR10H1	olfactory receptor, family 10, subfamily H, member 1	CCDS12335.1	chr19_15918238-15918238_A_C	204C>G	Substitution
Ependymoma	SE53PT	OR6B2	olfactory receptor, family 6, subfamily B, member 2	CCDS46559.1	chr2_240969456-240969456_G_A	131R>C	Substitution
Ependymoma	8129T	OSBPL7	oxysterol binding protein-like 7	CCDS11515.1	chr17 45890658-45890658 G A	571R>X	Substitution

Ependymoma	8129T	PAXIP1	PAX interacting (with transcription-activation domain) protein 1	CCDS47753.1	chr7_154752762-154752762_C_A	759A>S	Substitution
Ependymoma	CGLI 11	PAMR1	peptidase domain containing associated with muscle regeneration 1	CCDS7898.1	chr11_35414191-35414191_T_C	407K>R	Substitution
Ependymoma	7934T	PER3	period circadian clock 3	CCDS89.1	chr1 7887469-7887469 C A	819T>N	Substitution
Ependymoma	8648T	KCND2	potassium voltage-gated channel, Shal-related subfamily, member 2	CCDS5776.1	chr7_119915612-119915612_G_T	309G>V	Substitution
Ependymoma	SE53PT	PRDM15	PR domain containing 15	CCDS13676.1	chr21 43298949-43298949 C G	90A>P	Substitution
Ependymoma	SE53PT	PZP	pregnancy-zone protein	CCDS8600.1	chr12 9305502-9305502 A G	1347F>L	Substitution
Ependymoma	SE05PT	PAAF1	proteasomal ATPase-associated factor 1	ENST00000381783	chr5_38823301-38823301__G	NA	Insertion
Ependymoma	SE53PT	PTPRH	protein tyrosine phosphatase, receptor type, H	CCDS33110.1	chr19_55693151-55693151_C_T	1107A>T	Substitution
Ependymoma	SE01PT	RAB37	RAB37, member RAS oncogene family	CCDS32722.1	chr17_72733470-72733470_C_T	20P>L	Substitution
Ependymoma	8129T	RAP1GAP2	RAP1 GTPase activating protein 2	CCDS45573.1	chr17_2929708-2929708_G_A	644V>I	Substitution
Ependymoma	7692T	RASAL2	RAS protein activator like 2	CCDS1321.2	chr1 178359251-178359251 G T	158R>S	Substitution
Ependymoma	8648T	RCAN1	regulator of calcineurin 1	CCDS13637.1	chr21 35987240-35987240 G A	24A>V	Substitution
Ependymoma	7934T	RP1	retinitis pigmentosa 1 (autosomal dominant)	CCDS6160.1	chr8_55542255-55542255_G_A	1938R>H	Substitution
Ependymoma	SE03PT	RP1	retinitis pigmentosa 1 (autosomal dominant)	CCDS6160.1	chr8_55541706-55541706_G_A	1755R>K	Substitution
Ependymoma	SE53PT	RYR3	ryanodine receptor 3	CCDS45210.1	chr15 33936535-33936535 G T	1194G>C	Substitution
Ependymoma	8648T	SERPINB10	serpin peptidase inhibitor, clade B (ovalbumin), member 10	CCDS11990.1	chr18_61600419-61600419_C_A	257D>E	Substitution
Ependymoma	SE53PT	SIN3B	SIN3 transcription regulator homolog B (yeast)	CCDS32946.1	chr19_16962328-16962328_G_A	278V>M	Substitution
Ependymoma	SE01PT	STEAP1	six transmembrane epithelial antigen of the prostate 1	CCDS5614.1	chr7_89791240-89791240_A_G	204K>E	Substitution
Ependymoma	SE05PT	STEAP1	six transmembrane epithelial antigen of the prostate 1	CCDS5614.1	chr7_89790339-89790339_A_G	102H>R	Substitution
Ependymoma	SE01PT	SLX4	SLX4 structure-specific endonuclease subunit homolog (S. cerevisiae)	CCDS10506.2	chr16_3647687-3647687_C_T	459S>N	Substitution
Ependymoma	SE44PT	SCN7A	sodium channel, voltage-gated, type VII, alpha subunit	CCDS46442.1	chr2_167266809-167266809_G_A	1211T>M	Substitution
Ependymoma	8540T	SLC2A3	solute carrier family 2 (facilitated glucose transporter), member 3	CCDS8586.1	chr12_8083869-8083869_C_T	161G>D	Substitution
Ependymoma	8129T	SLC25A25	solute carrier family 25 (mitochondrial carrier; phosphate carrier), member 25	CCDS35151.1	chr9_130868463-130868463_T_C	312L>P	Substitution
Ependymoma	SE39PT	SLC25A36	solute carrier family 25 (pyrimidine nucleotide carrier), member 36	CCDS46927.1	chr3_140675460-140675460_G_C	45E>Q	Substitution
Ependymoma	SE52PT	SORBS1	sorbin and SH3 domain containing 1	CCDS31255.1	chr10 97135814-97135814 C G	NA	Substitution
Ependymoma	SE53PT	SYNE2	spectrin repeat containing, nuclear envelope 2	CCDS9761.2	chr14_64447420-64447420_C_T	540Q>X	Substitution
Ependymoma	SE39PT	SMPD1	sphingomyelin phosphodiesterase 1, acid lysosomal	CCDS44531.1	chr11_6411941-6411941_C_T	38A>V	Substitution
Ependymoma	CGLI 11	SPIRE1	spire homolog 1 (Drosophila)	CCDS32790.1	chr18 12525604-12525604 T C	NA	Substitution
Ependymoma	8648T	SND1	staphylococcal nuclease and tudor domain containing 1	CCDS34747.1	chr7_127326772-127326772_C_T	62R>W	Substitution
Ependymoma	8129T	TAOK1	TAO kinase 1	CCDS32601.1	chr17 27805358-27805358 A C	148I>L	Substitution
Ependymoma	SE07PT	TEP1	telomerase-associated protein 1	CCDS9548.1	chr14 20876412-20876412 T C	63K>E	Substitution
Ependymoma	8648T	TXNDC9	thioredoxin domain containing 9	CCDS2044.1	chr2 99936266-99936266 A	564-4delT	Deletion
Ependymoma	8129T	TCEB3B	transcription elongation factor B polypeptide 3B (elongin A2)	CCDS11932.1	chr18_44560420-44560420_C_T	406A>T	Substitution
Ependymoma	SE03PT	TMEM215	transmembrane protein 215	CCDS6530.1	chr9_32784829-32784829_C_G	216D>E	Substitution
Ependymoma	SE39PT	TCHH	trichohyalin	CCDS41396.1	chr1 152085191-152085191 G C	168R>G	Substitution
Ependymoma	SE01PT	TPPP	tubulin polymerization promoting protein	CCDS3856.1	chr5_678087-678087_C_T	30R>K	Substitution
Ependymoma	8527T	UQCRC2	ubiquinol-cytochrome c reductase core protein II	CCDS10601.1	chr16_21974108-21974108_A_G	139N>S	Substitution
Ependymoma	CGLI 11	USP18	ubiquitin specific peptidase 18	CCDS13752.1	chr22 17035970-17035970 C G	315D>E	Substitution
Ependymoma	SE39PT		Uncharacterized protein	ENST00000341750	chr19 56284090-56284090 G A	137A>T	Substitution
Ependymoma	SE39PT	UTS2R	urotensin 2 receptor	CCDS11810.1	chr17 80332927-80332927 C G	243R>G	Substitution
Ependymoma	SE52PT	ZC2HC1A	zinc finger, C2HC-type containing 1A	CCDS6223.1	chr8_79627496-79627496_G_T	249A>S	Substitution
Ependymoma	SE53PT	ZP3	zona pellucida glycoprotein 3 (sperm receptor)	CCDS47618.1	chr7_76071184-76071184__G	NA	Insertion
Myxopapillary	9625T	ACAT2	acetyl-CoA acetyltransferase 2	CCDS5268.1	chr6 160199281-160199281 T C	331I>T	Substitution
Myxopapillary	CGLI 25	ADRA1B	adrenergic; alpha-1B-; receptor	CCDS4347.1	chr5 159277283-159277283 C A	265L>I	Substitution
Myxopapillary	9625T	AP1M1	adaptor-related protein complex 1, mu 1 subunit	CCDS46008.1	chr19_16339014-16339014_A_C	307I>L	Substitution
Myxopapillary	9625T	C2	complement component 2	CCDS4728.1	chr6 31895915-31895915 G A	77R>Q	Substitution
Myxopapillary	CGLI 25	CDH5	cadherin 5; type 2 (vascular endothelium)	CCDS10804.1	chr16 64983781-64983781 G A	404S>N	Substitution
Myxopapillary	CGLI 25	ESX1	ESX homeobox 1	CCDS14516.1	chrX_103381846-103381846_T_G	314T>P	Substitution
Myxopapillary	8014T	FAM115C	family with sequence similarity 115, member C	CCDS34769.1	chr7_143031246-143031246_C_T	76P>S	Substitution
Myxopapillary	9625T	FAM163B	family with sequence similarity 163, member B	CCDS35171.1	chr9_136444273-136444273_C_G	124K>N	Substitution
Myxopapillary	8495T	IK	IK cytokine, down-regulator of HLA II	CCDS47280.1	chr5 140035574-140035574 G A	266M>I	Substitution
Myxopapillary	9625T	IQSEC1	IQ motif and Sec7 domain 1	NM 001134382	chr3 12942849-12942849 G T	993P>Q	Substitution
Myxopapillary	8014T	KIF4B	kinesin family member 4B	NM_001099293	chr5_154374102-154374102_C_T	164R>W	Substitution
Myxopapillary	8495T	MED13	mediator complex subunit 13	CCDS42366.1	chr17 60032845-60032845 A C	1956S>A	Substitution
Myxopapillary	8014T	MOSPD2	motile sperm domain containing 2	CCDS14162.1	chrX_14839308-14839308_T_A	244L>Q	Substitution
Myxopapillary	CGLI 25	MYOM2	myomesin (M-protein) 2; 165kDa	CCDS5957.1	chr8 2005012-2005012 T C	261L>P	Substitution
Myxopapillary	8543T	N/A					
Myxopapillary	8014T	OR2T5	olfactory receptor, family 2, subfamily T, member 5	CCDS31118.1	chr1_246718528-246718528_A_T	6R>W	Substitution
Myxopapillary	CGLI 13	PCSK7	proprotein convertase subtilisin/kexin type 7	CCDS8382.1	chr11 116582163-116582163 C A	706W>C	Substitution
Myxopapillary	CGLI 25	PKD1L2	polycystic kidney disease 1-like 2	CCDS42202.1	chr16 79781779-79781779 C A	555G>X	Substitution
Myxopapillary	8495T	PKHD1L1	polycystic kidney and hepatic disease 1 (autosomal recessive)-like 1	CCDS47911.1	chr8_110456960-110456960_C_T	1621P>L	Substitution
Myxopapillary	Ex-CGLI 25	POLR2E	polymerase (RNA) II (DNA directed) polypeptide E; 25kDa	CCDS12056.1	chr19_1041975-1041975_T_C	121M>V	Substitution
Myxopapillary	Ex-CGLI 25	RALYL	RNA-binding Raly-like protein isoform 1	NM 001100391	chr8 85937139-85937139 T C	169L>P	Substitution
Myxopapillary	9625T	RAPGEF3	Rap guanine nucleotide exchange factor (GEF) 3	CCDS41775.1	chr12_48151768-48151768_C_T	34V>M	Substitution
Myxopapillary	CGLI 25	RNMT	RNA (guanine-7-) methyltransferase	CCDS11867.1	chr18 13732491-13732491 C T	327R>C	Substitution

Myxopapillary	CGLI 25	RP1	retinitis pigmentosa 1 (autosomal dominant)	CCDS6160.1	chr8_55703949-55703949_C_T	1652R>C	Substitution
Myxopapillary	9625T	SFRP5	secreted frizzled-related protein 5	CCDS7472.1	chr10 99527287-99527287 G A	313A>V	Substitution
Myxopapillary	CGLI 25	SIPA1L1	signal-induced proliferation-associated 1 like 1	CCDS9807.1	chr14_71261222-71261222_A_G	1532T>A	Substitution
Myxopapillary	9625T	SRGAP3	SLIT-ROBO Rho GTPase activating protein 3	CCDS2572.1	chr3_9055187-9055187_T_A	651D>V	Substitution
Myxopapillary	CGLI 25	THUMPD2	THUMP domain containing 2	CCDS1805.1	chr2 39817590-39817590 T C	404D>G	Substitution
Myxopapillary	CGLI 25	TMEM117	transmembrane protein 117	CCDS8745.1	chr12 42524813-42524813 C T	31A>V	Substitution
Myxopapillary	CGLI 25	TMEM26	transmembrane protein 26	ENST00000399293	chr10 62845855-62845855 T G	213H>P	Substitution
Myxopapillary	CGLI 13	TTC16	tetratricopeptide repeat domain 16	CCDS6875.1	chr9_129526363-129526363_T_A	339V>E	Substitution
Myxopapillary	CGLI 25	UNC13C	protein unc-13 homolog C	NM 001080534	chr15 52574191-52574191 C T	1676P>L	Substitution
Subependymoma	1022013T	ABCA1	ATP-binding cassette, sub-family A (ABC1), member 1	CCDS6762.1	chr9_106627991-106627992_TG_	NA	Deletion
Subependymoma	8065T	ACADVL	acyl-CoA dehydrogenase, very long chain	ENST00000356839	chr17_7063980-7063994__GGGCGTGCAGGACGC	NA	Insertion
Subependymoma	1022013T	ADAM8	ADAM metallopeptidase domain 8	CCDS31319.1	chr10_134939024-134939024_C_T	35W>X	Substitution
Subependymoma	1022013T	ADAMTS17	ADAM metallopeptidase with thrombospondin type 1 motif, 17	CCDS10383.1	chr15_98512913-98512913_G_T	439F>L	Substitution
Subependymoma	1022013T	ADAMTSL1	ADAMTS-like 1	NM_001040272	chr9_18760757-18760757_G_T	792W>L	Substitution
Subependymoma	8065T	ADAMTSL 1	ADAMTS-like 1	NM_001040272	chr9_18711664-18711664_G_T	2006+1G>T	Substitution
Subependymoma	1022013T	ADAMTSL4	ADAMTS-like 4	CCDS955.1	chr1_148795424-148795424_C_T	512R>W	Substitution
Subependymoma	1022013T	AE000660.1-11	T cell receptor alpha locus	ENST00000390455	chr14_21661825-21661825__C	NA	Insertion
Subependymoma	1022013T	AGAP1	ArfGAP with GTPase domain, ankyrin repeat and PH domain 1	NM_001244888	chr2_236426154-236426154__CAGG	NA	Insertion
Subependymoma	1022013T	AGXT	alanine-glyoxylate aminotransferase	CCDS2543.1	chr2_241456987-241456987_C_T	11P>L	Substitution
Subependymoma	1022013T	AHNAK	AHNAK nucleoprotein	CCDS31584.1	chr11_62051300-62051300_G_A	2389P>S	Substitution
Subependymoma	1022013T	AK5	adenylate kinase 5	CCDS675.1	chr1_77796934-77796934__TAT	560S>SI	Insertion
Subependymoma	1022013T	ALDH4A1	aldehyde dehydrogenase 4 family, member A1	ENST00000375334	chr1_19081698-19081698_G_A	185T>I	Substitution
Subependymoma	1022013T	ALMS1	Alstrom syndrome 1	CCDS42697.1	chr2_73653772-73653772_C_G	3753L>V	Substitution
Subependymoma	SBP 8292014T	AMPD1	adenosine monophosphate deaminase 1	CCDS876.1	chr1 115018987-115018987 C T	570R>Q	Substitution
Subependymoma	1022013T	ANKRD42	ankyrin repeat domain 42	ENST00000260047	chr11_82636712-82636712_C_T	208R>X	Substitution
Subependymoma	1022013T	ANXA11	annexin A11	CCDS7364.1	chr10_81911746-81911746_C_T	369E>K	Substitution
Subependymoma	1022013T	APOL5	apolipoprotein L, 5	CCDS13920.1	chr22_34454756-34454756_G_T	389Q>H	Substitution
Subependymoma	1022013T	ARAP1	ArfGAP with RhoGAP domain, ankyrin repeat and PH domain 1	ENST00000340247	chr11_72101371-72101371_C_A	2C>F	Substitution
Subependymoma	1022013T	ARFGAP1	ADP-ribosylation factor GTPase activating protein 1	CCDS13516.1	chr20_61378356-61378356_C_T	84R>X	Substitution
Subependymoma	1022013T	ARHGEF4	Rho guanine nucleotide exchange factor (GEF) 4	CCDS2165.1	chr2_131520105-131520105_C_T	649T>M	Substitution
Subependymoma	1022013T	ARID1B	AT rich interactive domain 1B (SWI1-like)	CCDS5251.1	chr6_157570602-157570602_G_A	2194R>H	Substitution
Subependymoma	SE42PT	ARID1B	AT rich interactive domain 1B (SWI1-like)	CCDS5251.1	chr6 157522292-157522292 CT	NA	Insertion
Subependymoma	SE42PT	ARID1B	AT rich interactive domain 1B (SWI1-like)	CCDS5251.1	chr6 157522294-157522294 T A	NA	Substitution
Subependymoma	1022013T	ARVCF	armadillo repeat gene deleted in velocardiofacial syndrome	CCDS13771.1	chr22_18345108-18345108_G_A	567S>L	Substitution
Subependymoma	1022013T	ASCC3	activating signal cointegrator 1 complex subunit 3	CCDS5046.1	chr6_101072723-101072723_G_A	1931A>V	Substitution
Subependymoma	1022013T	ASXL2	additional sex combs like 2 (Drosophila)	NM_018263	chr2_25818880-25818880_A_G	1277V>A	Substitution
Subependymoma	1022013T	AXDND1	axonemal dynein light chain domain containing 1	CCDS30948.1	chr1_177770660-177770662_GAA_	NA	Deletion
Subependymoma	1022013T	BANP	BTG3 associated nuclear protein	ENST00000286122	chr16_86566214-86566214__T	NA	Insertion
Subependymoma	1022013T	BC037579	-	ENST00000398122	chr16_72952155-72952155_C_A	34R>L	Substitution
Subependymoma	1022013T	BC064385	uncharacterized protein	ENST00000409982	chr2_131050856-131050856_G_A	114A>T	Substitution
Subependymoma	1022013T	BCL2L11	BCL2-like 11 (apoptosis facilitator)	NM_001204113	chr2_111616425-111616425_G_A	93W>X	Substitution
Subependymoma	1022013T	BCL7B	B-cell CLL/lymphoma 7B	CCDS5550.1	chr7_72592268-72592268_C_G	106D>H	Substitution
Subependymoma	1022013T	BCOR	BCL6 corepressor	CCDS14250.1	chrX_39807868-39807868_C_A	1228A>S	Substitution
Subependymoma	1022013T	BCORL1	BCL6 corepressor-like 1	CCDS14616.1	chrX_128974709-128974709_G_A	94D>N	Substitution
Subependymoma	1022013T	BDP1	B double prime 1, subunit of RNA polymerase III transcription initiation factor IIIB	CCDS43328.1	chr5_70842277-70842277_A_C	1201E>A	Substitution
Subependymoma	1022013T	BEST2	bestrophin 2	CCDS42506.1	chr19_12727555-12727557_TTC_	281F>-	Deletion
Subependymoma	8065T	BHMT2	betaine--homocysteine S-methyltransferase 2	CCDS4045.1	chr5_78414948-78414948_C_G	259P>R	Substitution
Subependymoma	1022013T	BV17S1J1.1	UNVERIFIED: T cell receptor beta variable 19	ENST00000390393	chr7_142008010-142008010_G_A	106A>T	Substitution
Subependymoma	1022013T	C12orf56	chromosome 12 open reading frame 56	NM_001170633	chr12_62998814-62998814__GTT	234N>KT	Insertion
Subependymoma	1022013T	C14orf159 variant protein	chromosome 14 open reading frame 159	ENST00000298858	chr14_90703668-90703668_G_A	120W>X	Substitution
Subependymoma	1022013T	C15orf65	chromosome 15 open reading frame 65	NM_001198784	chr15_53497989-53497989_A_G	99N>D	Substitution
Subependymoma	1022013T	C17orf50	chromosome 17 open reading frame 50	CCDS42298.1	chr17_31115153-31115153_T_A	10L>M	Substitution
Subependymoma	1022013T	C21orf123	COL18A1 antisense RNA 1	ENST00000330082	chr21_45666844-45666844_C_T	22A>T	Substitution
Subependymoma	1022013T	C22orf31	chromosome 22 open reading frame 31	CCDS13848.1	chr22_27784819-27784819_G_A	262Q>X	Substitution
Subependymoma	1022013T	C2orf78	chromosome 2 open reading frame 78	NM_001080474	chr2_73897493-73897493_C_A	879R>S	Substitution
Subependymoma	1022013T	C9orf173	chromosome 9 open reading frame 173	NM_001256699	chr9_139267406-139267406_C_A	277S>R	Substitution
Subependymoma	1022013T	C9orf43	chromosome 9 open reading frame 43	CCDS6796.1	chr9_115227467-115227467__GCA	296R>RQ	Insertion
Subependymoma	9521T	CADM2	cell adhesion molecule 2	CCDS33792.1	chr3_86197569-86197569_A_T	398K>N	Substitution
Subependymoma	8065T	CAP1	CAP, adenylate cyclase-associated protein 1 (yeast)	CCDS41309.1	chr1_40305883-40305883_C_T	239P>S	Substitution
Subependymoma	1022013T	CASKIN2	CASK interacting protein 2	CCDS11723.1	chr17_71012725-71012725_G_A	319R>C	Substitution
Subependymoma	1022013T	CCDC114	coiled-coil domain containing 114	CCDS12714.1	chr19_53499022-53499022_C_T	41A>T	Substitution
Subependymoma	1022013T	CCDC14	coiled-coil domain containing 14	CCDS3025.1	chr3_125116741-125116741_T_G	653N>H	Substitution
Subependymoma	1022013T	CCDC155	coiled-coil domain containing 155	NM_144688	chr19_54612230-54612230_C_T	481P>L	Substitution
Subependymoma	1022013T	CCDC171	coiled-coil domain containing 171	ENST00000359391	chr9_15912172-15912172_T_C	127C>R	Substitution
Subependymoma	1022013T	CCDC178	coiled-coil domain containing 178	CCDS42424.1	chr18_29231155-29231155_T_A	25E>V	Substitution
Subependymoma	1022013T	CCDC33	coiled-coil domain containing 33	CCDS42058.1	chr15_72323457-72323459_AAG_	NA	Deletion
Subependymoma	1022013T	CCDC88C	coiled-coil domain containing 88C	NM_001080414	chr14_90819477-90819477_T_C	1527T>A	Substitution
Subependymoma	1022013T	CD5	CD5 molecule	CCDS8000.1	chr11_60642999-60642999_C_T	146P>L	Substitution
Subependymoma	1022013T	CDC37	cell division cycle 37	CCDS12237.1	chr19_10365009-10365009_C_T	279E>K	Substitution
Subependymoma	1022013T	CDKL1	cyclin-dependent kinase-like 1 (CDC2-related kinase)	ENST00000356146	chr14_49946981-49946981_T_C	58K>E	Substitution
Subependymoma	1022013T	CEACAM19	carcinoembryonic antigen-related cell adhesion molecule 19	CCDS12641.1	chr19_49875443-49875443_G_T	235A>S	Substitution
Subependymoma	1022013T	CECR2	cat eye syndrome chromosome region, candidate 2	ENST00000400585	chr22_16402114-16402114_G_A	739R>Q	Substitution

Subependymoma	1022013T	CELA1	chymotrypsin-like elastase family, member 1	CCDS8812.1	chr12_50009866-50009866__G	NA	Insertion
Subependymoma	1022013T	CEP164	centrosomal protein 164kDa	CCDS31683.1	chr11_116737915-116737915_T_A	183M>K	Substitution
Subependymoma	1022013T	CHAT	choline O-acetyltransferase	CCDS7232.1	chr10_50527556-50527556_G_A	NA	Substitution
Subependymoma	1022013T	CHIT1	chitinase 1 (chitotriosidase)	CCDS1436.1	chr1_201453598-201453621__AGACCATGGCCCCGCCCAGTCCCT	NA	Insertion
Subependymoma	1022013T	CHRNA5	cholinergic receptor, nicotinic, alpha 5 (neuronal)	CCDS10304.1	chr15_76669310-76669311_CA_	NA	Deletion
Subependymoma	1022013T	CHRNB3	cholinergic receptor, nicotinic, beta 3 (neuronal)	CCDS6134.1	chr8_42706298-42706298_C_T	231R>C	Substitution
Subependymoma	1022013T	CLASP2	cytoplasmic linker associated protein 2	NM_015097	chr3_33608971-33608971_G_A	664S>L	Substitution
Subependymoma	1022013T	CLIP1	CAP-GLY domain containing linker protein 1	CCDS9232.1	chr12_121378872-121378872_A_T	963F>L	Substitution
Subependymoma	1022013T	CLN6	ceroid-lipofuscinosis, neuronal 6, late infantile, variant	CCDS10227.1	chr15_66287545-66287545_C_G	308S>T	Substitution
Subependymoma	1022013T	CNPY4	canopy FGF signaling regulator 4	CCDS34701.1	chr7_99557938-99557938_G_T	80S>I	Substitution
Subependymoma	1022013T	COL23A1	collagen, type XXIII, alpha 1	CCDS4436.1	chr5_177614553-177614553__T	NA	Insertion
Subependymoma	1022013T	CPA6	carboxypeptidase A6	CCDS6200.1	chr8_68508936-68508936_C_T	311R>Q	Substitution
Subependymoma	1022013T	CPEB3	cytoplasmic polyadenylation element binding protein 3	CCDS31246.1	chr10_93802019-93802019_C_T	676R>Q	Substitution
Subependymoma	1022013T	CPN1	carboxypeptidase N, polypeptide 1	CCDS7486.1	chr10_101819504-101819504_C_T	178G>D	Substitution
Subependymoma	1022013T	CPS1	carbamoyl-phosphate synthase 1, mitochondrial	NM_001122633	chr2_211129698-211129698__TCT	5I>IF	Insertion
Subependymoma	1022013T	CTNNBL1	catenin, beta like 1	CCDS13298.1	chr20_35808333-35808333_G_C	126V>L	Substitution
Subependymoma	1022013T	CTSH	cathepsin H	CCDS10308.1	chr15_77004727-77004727_T_C	NA	Substitution
Subependymoma	1022013T	CUL9	cullin 9	CCDS4890.1	chr6_43292040-43292040_G_A	2035A>T	Substitution
Subependymoma	1022013T	CYLC1	cylicin, basic protein of sperm head cytoskeleton 1	CCDS35341.1	chrX_83015126-83015126_G_A	252G>R	Substitution
Subependymoma	1022013T	CYLC1	cylicin, basic protein of sperm head cytoskeleton 1	CCDS35341.1	chrX_83015845-83015845_G_T	491E>D	Substitution
Subependymoma	1022013T	CYP1B1	cytochrome P450, family 1, subfamily B, polypeptide 1	CCDS1793.1	chr2_38155894-38155894_G_C	48R>G	Substitution
Subependymoma	1022013T	CYP2W1	cytochrome P450, family 2, subfamily W, polypeptide 1	CCDS5319.2	chr7_993647-993647_G_A	366R>H	Substitution
Subependymoma	1022013T	CYP4A11	cytochrome P450, family 4, subfamily A, polypeptide 11	CCDS543.1	chr1_47168409-47168409_G_A	509L>F	Substitution
Subependymoma	1022013T	DCHS1	dachsous cadherin-related 1	CCDS7771.1	chr11_6599770-6599770_G_A	3238P>L	Substitution
Subependymoma	1022013T	DCHS1	dachsous cadherin-related 1	CCDS7771.1	chr11_6618820-6618820_C_T	201G>R	Substitution
Subependymoma	1022013T	DDHD1	DDHD domain containing 1	CCDS9714.1	chr14_52689431-52689431_C_T	46G>S	Substitution
Subependymoma	1022013T	DIDO1	death inducer-obliterator 1	CCDS33506.1	chr20_60983880-60983891_TGTGGCTGCTGT_	NA	Deletion
Subependymoma	1022013T	DIP2C	DIP2 disco-interacting protein 2 homolog C (Drosophila)	ENST00000402953	chr10_520774-520774_C_T	91R>Q	Substitution
Subependymoma	1022013T	DMRTA2	DMRT-like family A2	NM_032110	chr1_50657123-50657123_C_T	477S>N	Substitution
Subependymoma	1022013T	DNAH6	dynein, axonemal, heavy chain 6	NM_001370	chr2_84889107-84889107_G_A	3890R>H	Substitution
Subependymoma	1022013T	DNAJC28	DnaJ (Hsp40) homolog, subfamily C, member 28	CCDS13626.1	chr21_33782627-33782631_TTAAA_	NA	Deletion
Subependymoma	1022013T	DNAJC4	DnaJ (Hsp40) homolog, subfamily C, member 4	CCDS41666.1	chr11_63756505-63756505_C_T	70P>S	Substitution
Subependymoma	1022013T	DOK3	docking protein 3	NM_001144875	chr5_176862781-176862783_GAG_	NA	Deletion
Subependymoma	1022013T	DPP9	dipeptidyl-peptidase 9	NM_139159	chr19_4640635-4640635_C_T	566E>K	Substitution
Subependymoma	1022013T	DSP	desmoplakin	CCDS4501.1	chr6_7487148-7487148__A	NA	Insertion
Subependymoma	1022013T	DST	dystonin	NM_001144769	chr6_56449081-56449081_G_A	5111A>V	Substitution
Subependymoma	1022013T	E2F7	E2F transcription factor 7	CCDS9016.1	chr12_75946010-75946010_T_C	642M>V	Substitution
Subependymoma	1022013T	EBLN1	endogenous Bornavirus-like nucleoprotein 1	NM_001199938	chr10_22538491-22538491__AGA	143L>LL	Insertion
Subependymoma	1022013T	EFCAB4A	EF-hand calcium binding domain 4A	ENST00000327417	chr11_820602-820602__GGTC	NA	Insertion
Subependymoma	1022013T	EHMT2	euchromatic histone-lysine N-methyltransferase 2	CCDS4725.1	chr6_31972739-31972739_C_G	17A>P	Substitution
Subependymoma	1022013T	EIF2AK4	eukaryotic translation initiation factor 2 alpha kinase 4	CCDS42016.1	chr15_38056302-38056307__CGACGA	NA	Insertion
Subependymoma	1022013T	ENSG00000212673	uncharacterized protein	ENST00000391369	chr1_32152586-32152596_TTTA_	NA	Deletion
Subependymoma	1022013T	ENSG00000214279	-	ENST00000356567	chr10_135128247-135128253 TGTGGGG	NA	Deletion
Subependymoma	1022013T	EPHB4	EPH receptor B4	CCDS5706.1	chr7_100240808-100240808_C_T	917R>Q	Substitution
Subependymoma	SE42PT	ESX1	ESX homeobox 1	CCDS14516.1	chrX 103495090-103495090 G C	347P>R	Substitution
Subependymoma	1022013T	EVI2A	ecotropic viral integration site 2A	CCDS32608.1	chr17_26669815-26669815_T_A	138K>X	Substitution
Subependymoma	1022013T	EXD1	exonuclease 3'-5' domain containing 1	CCDS10072.1	chr15_39264017-39264017_T_C	317K>E	Substitution
Subependymoma	1022013T	F2RL2	coagulation factor II (thrombin) receptor-like 2	CCDS4031.1	chr5_75949705-75949705_T_C	195I>V	Substitution
Subependymoma	1022013T	FAM120C	family with sequence similarity 120C	CCDS14356.1	chrX_54226043-54226048__GGCGGC	NA	Insertion
Subependymoma	1022013T	FAM200A	family with sequence similarity 200, member A	CCDS5668.1	chr7_98983358-98983358_C_T	203W>X	Substitution
Subependymoma	1022013T	FBLN7	fibulin 7	CCDS2095.1	chr2_112633760-112633760_C_T	38R>C	Substitution
Subependymoma	1022013T	FBRSL1	fibrosin-like 1	NM_001142641	chr12_131612405-131612405_G_A	168V>M	Substitution
Subependymoma	1022013T	FBXO41	F-box protein 41	NM_001080410	chr2_73347118-73347118_G_T	369P>H	Substitution
Subependymoma	1022013T	FCN2	ficolin (collagen/fibrinogen domain containing lectin) 2	CCDS6983.1	chr9_136918847-136918847_C_T	236T>M	Substitution
Subependymoma	1022013T	FKBP15	FK506 binding protein 15, 133kDa	NM_015258	chr9_114971972-114971972__TTC	946E>EK	Insertion
Subependymoma	1022013T	FNBP4	formin binding protein 4	CCDS41644.1	chr11_47745307-47745307_G_C	37T>S	Substitution
Subependymoma	1022013T	FOXC1	forkhead box C1	CCDS4473.1	chr6_1557017-1557017__CGG	446H>HG	Insertion
Subependymoma	1022013T	FTCD	formimidoyltransferase cyclodeaminase	CCDS13731.1	chr21_46390268-46390268__C	NA	Insertion
Subependymoma	1022013T	FUK	fucokinase	CCDS10891.2	chr16_69064715-69064715_C_T	579R>C	Substitution
Subependymoma	1022013T	FZD1	frizzled family receptor 1	CCDS5620.1	chr7_90732396-90732396__CCG	89->P	Insertion
Subependymoma	1022013T	GAS6	growth arrest-specific 6	NM_000820	chr13_113589949-113589949_C_T	NA	Substitution
Subependymoma	1022013T	GDPD4	glycerophosphodiester phosphodiesterase domain containing 4	CCDS8249.1	chr11_76632437-76632437__A	NA	Insertion
Subependymoma	1022013T	GINS2	GINS complex subunit 2 (Psf2 homolog)	CCDS10953.1	chr16_84269366-84269366_G_T	171R>S	Substitution
Subependymoma	1022013T	GMEB1	glucocorticoid modulatory element binding protein 1	CCDS327.1	chr1_28913814-28913814_A_G	555E>G	Substitution
Subependymoma	1022013T	GPR33	G protein-coupled receptor 33 (gene/pseudogene)	NM_001197184	chr14_31022498-31022498_G_C	142P>R	Substitution
Subependymoma	1022013T	GRXCR1	glutaredoxin, cysteine rich 1	CCDS43225.1	chr4_42590153-42590153_C_T	38P>L	Substitution
Subependymoma	1022013T	GSC	goosecoid homeobox	CCDS9930.1	chr14_94305958-94305958__CGC	50->A	Insertion
Subependymoma	1022013T	GSG2	germ cell associated 2 (haspin)	CCDS11036.1	chr17_3574096-3574096_C_T	40R>W	Substitution
Subependymoma	1022013T	GTF2B	general transcription factor IIB	CCDS715.1	chr1 89125568-89125568 C T	30G>S	Substitution
Subependymoma	1022013T	GUCY2D	guanylate cyclase 2D, membrane (retina-specific)	CCDS11127.1	chr17_7853600-7853600_C_T	574R>C	Substitution
Subependymoma	SE42PT	HCAR3	hydroxycarboxylic acid receptor 3	NM 006018	chr12 123200354-123200354 G A	311R>C	Substitution
Subependymoma	1022013T	HDGFRP2	hepatoma-derived growth factor-related protein 2 isoform 1	CCDS42472.1	chr19_4452243-4452243_G_C	615K>N	Substitution

Subependymoma	1022013T	HIF3A	hypoxia inducible factor 3, alpha subunit	ENST00000244303	chr19_51507544-51507547_TATT_	NA	Deletion
Subependymoma	1022013T	HOMEZ	homeobox and leucine zipper encoding	NM_020834	chr14_22814666-22814666_T_A	537E>D	Substitution
Subependymoma	1022013T	HOXC13	homeobox C13	CCDS8865.1	chr12_52619001-52619001__TTA	15L>LI	Insertion
Subependymoma	1022013T	HOXC9	homeobox C9	CCDS8869.1	chr12_52680298-52680298_A_T	20N>I	Substitution
Subependymoma	1022013T	HS6ST1	heparan sulfate 6-O-sulfotransferase 1	CCDS42748.1	chr2_128792486-128792486_C_G	41S>T	Substitution
Subependymoma	SBP 8292014T	HS6ST1	heparan sulfate 6-O-sulfotransferase 1	CCDS42748.1	chr2_128742697-128742697_G_T	249R>S	Substitution
Subependymoma	1022013T	IGLV5-45	UNVERIFIED: immunoglobulin lambda variable 5-45	ENST00000390296	chr22_21060696-21060696_C_T	64P>L	Substitution
Subependymoma	1022013T	INADL	InaD-like (Drosophila)	CCDS617.2	chr1_62152887-62152887_G_C	1178G>A	Substitution
Subependymoma	1022013T	INSC	inscuteable homolog (Drosophila)	CCDS41621.1	chr11_15127289-15127289_G_A	45G>E	Substitution
Subependymoma	1022013T	IPO8	importin 8	CCDS8719.1	chr12_30706684-30706684_C_G	533K>N	Substitution
Subependymoma	1022013T	IRF7	interferon regulatory factor 7	CCDS7705.1	chr11_603781-603781_T_	NA	Deletion
Subependymoma	1022013T	ITGA11	integrin, alpha 11	NM_001004439	chr15_66411762-66411762_G_A	512R>X	Substitution
Subependymoma	1022013T	ITGA2	integrin, alpha 2 (CD49B, alpha 2 subunit of VLA-2 receptor)	CCDS3957.1	chr5_52412172-52412172_C_A	1001N>K	Substitution
Subependymoma	1022013T	ITGB5	integrin, beta 5	CCDS3030.1	chr3_125965193-125965201_CTTCTTGAA	NA	Deletion
Subependymoma	1022013T	JPH3	junctophilin 3	NM_001271604	chr16_86195395-86195395__CTG	144P>PA	Insertion
Subependymoma	1022013T	KBTBD13	kelch repeat and BTB (POZ) domain containing 13	NM_001101362	chr15_63156208-63156208_T_G	1M>R	Substitution
Subependymoma	1022013T	KCNH2	potassium voltage-gated channel, subfamily H (eag-related), member 2	NM_172056	chr7_150278077-150278077_C_A	837G>V	Substitution
Subependymoma	1022013T	KCNQ3	potassium voltage-gated channel, KQT-like subfamily, member 3	CCDS34943.1	chr8_133244918-133244918_T_C	414E>G	Substitution
Subependymoma	1022013T	KIAA0947	-	NM_015325	chr5_5517935-5517935_G_T	1830D>Y	Substitution
Subependymoma	1022013T	KIAA1551	-	CCDS8725.2	chr12_32029057-32029057_G_A	1301E>K	Substitution
Subependymoma	1022013T	KIAA1751	-	NM_001080484	chr1_1889967-1889967__CTC	404K>KR	Insertion
Subependymoma	1022013T	KIR3DL1	killer cell immunoglobulin-like receptor, three domains, long cytoplasmic tail, 1	CCDS42621.1	chr19_60023126-60023126_G_A	168V>I	Substitution
Subependymoma	1022013T	KISS1	KiSS-1 metastasis-suppressor	CCDS41454.1	chr1_202426235-202426235_T_	NA	Deletion
Subependymoma	1022013T	KLK9	kallikrein-related peptidase 9	CCDS12816.1	chr19_56204251-56204251_G_A	67P>L	Substitution
Subependymoma	1022013T	KMT2C	lysine (K)-specific methyltransferase 2C	CCDS5931.1	chr7_151576189-151576189_G_A	755Q>X	Substitution
Subependymoma	1022013T	KMT2D	lysine (K)-specific methyltransferase 2D	NM_003482	chr12_47718828-47718828_G_A	2860R>C	Substitution
Subependymoma	1022013T	KRT24	keratin 24	CCDS11372.1	chr17_36111661-36111661_A_	NA	Deletion
Subependymoma	1022013T	KRTAP6-2	keratin associated protein 6-2	CCDS13600.1	chr21_30893058-30893058_C_T	3G>S	Substitution
Subependymoma	1022013T	LAMP2	lysosomal-associated membrane protein 2	CCDS14599.1	chrX_119473400-119473400_A_C	79C>W	Substitution
Subependymoma	1022013T	LAPTM4B	lysosomal protein transmembrane 4 beta	ENST00000378722	chr8_98857104-98857104_G_A	35R>Q	Substitution
Subependymoma	1022013T	LEPRE1	leucine proline-enriched proteoglycan (leprecan) 1	CCDS472.1	chr1_43004804-43004804_C_A	142K>N	Substitution
Subependymoma	1022013T	LFNG	LFNG O-fucosylpeptide 3-beta-N-acetylglucosaminyltransferase	CCDS34587.1	chr7_2533044-2533044_G_A	346V>M	Substitution
Subependymoma	1022013T	LOC285908	UNVERIFIED: long intergenic non-protein coding RNA 174	ENST00000324866	chr7_65479979-65479979__AT	NA	Insertion
Subependymoma	1022013T	LOC286238	uncharacterized protein	NM_001100111	chr9_90452164-90452164__TGG	100H>PN	Insertion
Subependymoma	1022013T	LRBA	LPS-responsive vesicle trafficking, beach and anchor containing	CCDS3773.1	chr4_151426630-151426630_G_A	2686T>I	Substitution
Subependymoma	1022013T	LRRC36	leucine rich repeat containing 36	CCDS32467.1	chr16_65976329-65976329_G_A	701G>S	Substitution
Subependymoma	1022013T	LRRC43	leucine rich repeat containing 43	CCDS41854.1	chr12_121253913-121253913_G_A	463E>K	Substitution
Subependymoma	1022013T	LRRC7	leucine rich repeat containing 7	CCDS645.1	chr1_70277766-70277766_C_T	1186A>V	Substitution
Subependymoma	1022013T	LRRIQ1	leucine-rich repeats and IQ motif containing 1	CCDS41816.1	chr12_83983282-83983282_A_G	835S>G	Substitution
Subependymoma	1022013T	LSM5	LSM5 homolog, U6 small nuclear RNA associated (S. cerevisiae)	CCDS5438.1	chr7_32495400-32495400_A_C	43F>C	Substitution
Subependymoma	1022013T	LTBP1	latent transforming growth factor beta binding protein 1	CCDS33177.1	chr2_33439264-33439264_A_G	1367N>S	Substitution
Subependymoma	1022013T	LXN	latexin	CCDS3183.1	chr3_159871445-159871445_G_T	63Q>K	Substitution
Subependymoma	1022013T	MEOX2	mesenchyme homeobox 2	CCDS34605.1	chr7_15692313-15692313_A_C	80H>Q	Substitution
Subependymoma	1022013T	MFSD2A	major facilitator superfamily domain containing 2A	CCDS446.1	chr1_40207814-40207814_C_T	527A>V	Substitution
Subependymoma	1022013T	MICA	MHC class I polypeptide-related sequence A	NM_000247	chr6_31487981-31487981_G_	NA	Deletion
Subependymoma	1022013T	MIIP	migration and invasion inhibitory protein	CCDS143.1	chr1_12005087-12005087_G_C	NA	Substitution
Subependymoma	1022013T	MKI67	marker of proliferation Ki-67	CCDS7659.1	chr10_129794629-129794629_C_T	1822R>H	Substitution
Subependymoma	1022013T	MLYCD	malonyl-CoA decarboxylase	CCDS42206.1	chr16_82503405-82503405_A_G	294T>A	Substitution
Subependymoma	1022013T	MMEL1	membrane metallo-endopeptidase-like 1	CCDS30569.1	chr1_2513272-2513272_C_T	699D>N	Substitution
Subependymoma	1022013T	MMP16	matrix metallopeptidase 16 (membrane-inserted)	CCDS43752.1	chr8_89150851-89150851__A	NA	Insertion
Subependymoma	1022013T	MRPS34	mitochondrial ribosomal protein S34	CCDS10444.1	chr16_1762799-1762799__ACCT	NA	Insertion
Subependymoma	1022013T	MS4A15	membrane-spanning 4-domains, subfamily A, member 15	NM_001098835	chr11_60291594-60291594_C_T	80L>F	Substitution
Subependymoma	1022013T	MTR	5-methyltetrahydrofolate-homocysteine methyltransferase	CCDS1614.1	chr1_235082974-235082974_A_	NA	Deletion
Subependymoma	1022013T	MTRR	5-methyltetrahydrofolate-homocysteine methyltransferase reductase	CCDS3874.1	chr5_7939760-7939762_TGT_	391C>-	Deletion
Subependymoma	1022013T	MUC16	mucin 16, cell surface associated	NM_024690	chr19_8921439-8921439_T_A	9003T>S	Substitution
Subependymoma	1022013T	MUC6	mucin 6, oligomeric mucus/gel-forming	NM_005961	chr11_1021059-1021059__G	NA	Insertion
Subependymoma	1022013T	MYO16	myosin XVI	CCDS32008.1	chr13_108590826-108590826_C_T	1400A>V	Substitution
Subependymoma	1022013T	MYO7A	myosin VIIA	NM_000260	chr11_76545579-76545579_C_T	206R>C	Substitution
Subependymoma	1022013T	MYO7A	myosin VIIA	NM_001127179	chr11_76573420-76573441_GAGGCGGGGACACCAGGGCCTG_	NA	Deletion
Subependymoma	1022013T	MYO9A	myosin IXA	CCDS10239.1	chr15_69959906-69959906_C_T	1816G>S	Substitution
Subependymoma	1022013T	MYOF	myoferlin	CCDS41551.1	chr10_95149177-95149177_C_T	395V>M	Substitution
Subependymoma	1022013T	MYOM1	myomesin 1	NM_003803	chr18_3119368-3119368_T_C	886S>G	Substitution
Subependymoma	1022013T	NADK	NAD kinase	NM_001198995	chr1_1679688-1679688_G_A	48T>M	Substitution
Subependymoma	1022013T	NAGS	N-acetylglutamate synthase	CCDS11473.1	chr17_39441432-39441432__G	NA	Insertion
Subependymoma	1022013T	NBN	nibrin	CCDS6249.1	chr8_91059697-91059697_T_C	171I>V	Substitution
Subependymoma	1022013T	NCCRP1	non-specific cytotoxic cell receptor protein 1 homolog (zebrafish)	CCDS12529.1	chr19_44381717-44381717_G_A	174E>K	Substitution
Subependymoma	1022013T	NECAB1	N-terminal EF-hand calcium binding protein 1	NM_022351	chr8_92022253-92022253_G_T	271A>S	Substitution
Subependymoma	1022013T	NFRKB	nuclear factor related to kappaB binding protein	CCDS8483.1	chr11_129248164-129248164_G_A	888T>M	Substitution
Subependymoma	1022013T	NKX3-2	NK3 homeobox 2	CCDS3410.1	chr4_13154902-13154902_T_A	79T>S	Substitution
Subependymoma	1022013T	NLRC5	NLR family, CARD domain containing 5	CCDS10773.1	chr16_55618334-55618334_C_T	660H>Y	Substitution
Subependymoma	1022013T	NLRP1	NLR family, pyrin domain containing 1	CCDS42246.1	chr17_5364953-5364953_C_T	1296G>D	Substitution
Subependymoma	1022013T	NNMT	nicotinamide N-methyltransferase	CCDS8368.1	chr11_113672530-113672530_T_A	14H>Q	Substitution
Subependymoma	1022013T	NOD2	nucleotide-binding oligomerization domain containing 2	CCDS10746.1	chr16_49314041-49314041_G_C	908G>R	Substitution

Subependymoma	1022013T	NRD1	nardilysin (N-arginine dibasic convertase)	CCDS559.1	chr1_52078663-52078665_TTC_	NA	Deletion
Subependymoma	1022013T	NRSN1	neurensin 1	CCDS4549.1	chr6_24253976-24253976_G_	NA	Deletion
Subependymoma	1022013T	NTN5	netrin 5	CCDS33068.1	chr19_53865563-53865563_G_A	165R>C	Substitution
Subependymoma	1022013T	NUPL1	nucleoporin like 1	CCDS9314.1	chr13_24803676-24803676_A_G	472Q>R	Substitution
Subependymoma	1022013T	NWD1	NACHT and WD repeat domain containing 1	CCDS32945.1	chr19_16745101-16745101_C_T	724P>S	Substitution
Subependymoma	1022013T	OR13A1	olfactory receptor, family 13, subfamily A, member 1	CCDS31188.1	chr10_45119072-45119072__A	NA	Insertion
Subependymoma	1022013T	OR1B1	olfactory receptor, family 1, subfamily B, member 1	CCDS35126.1	chr9_124431592-124431592__A	NA	Insertion
Subependymoma	1022013T	OR2T2	olfactory receptor, family 2, subfamily T, member 2	CCDS31116.1	chr1_246683213-246683214_TA_	NA	Deletion
Subependymoma	1022013T	OR5B3	olfactory receptor, family 5, subfamily B, member 3	CCDS31549.1	chr11_57927369-57927369_G_	NA	Deletion
Subependymoma	1022013T	OR6K2	olfactory receptor, family 6, subfamily K, member 2	CCDS30902.1	chr1_156936714-156936714_G_A	118A>V	Substitution
Subependymoma	1022013T	OR7G3	olfactory receptor, family 7, subfamily G, member 3	CCDS32899.1	chr19_9097699-9097699__ATGGT	NA	Insertion
Subependymoma	1022013T	OTOG	otogelin	NM_001277269	chr11_17588804-17588804_G_A	1806R>H	Substitution
Subependymoma	1022013T	OXR1	oxidation resistance 1	CCDS6304.1	chr8_107774156-107774156_G_T	96A>S	Substitution
Subependymoma	SBP 8292014T	PCMTD1	protein-L-isoaspartate (D-aspartate) O-methyltransferase domain containing 1	CCDS6148.1	chr8_52895784-52895784_G_A	252R>X	Substitution
Subependymoma	1022013T	PCNT	pericentrin	CCDS33592.1	chr21_46578877-46578877_G_A	136G>S	Substitution
Subependymoma	1022013T	PDE12	phosphodiesterase 12	CCDS33772.1	chr3_57518416-57518416_G_A	424A>T	Substitution
Subependymoma	1022013T	PEBP4	phosphatidylethanolamine-binding protein 4	CCDS43724.1	chr8_22626847-22626847_G_A	222A>V	Substitution
Subependymoma	1022013T	PEG10	paternally expressed 10	NM_001172438	chr7_94130815-94130815_G_A	80R>Q	Substitution
Subependymoma	1022013T	PEX2	peroxisomal biogenesis factor 2	CCDS6221.1	chr8_78058338-78058338_A_G	211I>T	Substitution
Subependymoma	1022013T	PIEZO2	piezo-type mechanosensitive ion channel component 2	NM_022068	chr18_10847168-10847168_A_C	178Y>X	Substitution
Subependymoma	1022013T	PIGO	phosphatidylinositol glycan anchor biosynthesis, class O	CCDS6575.1	chr9_35085102-35085102_G_T	154A>D	Substitution
Subependymoma	1022013T	PITPNM2	phosphatidylinositol transfer protein, membrane-associated 2	CCDS9242.1	chr12_122051336-122051336_C_T	389R>H	Substitution
Subependymoma	1022013T	PITRM1	pitrilysin metallopeptidase 1	NM_001242307	chr10_3183558-3183570_AAAAAAGAAACAA_	NA	Deletion
Subependymoma	1022013T	PLEKHH3	pleckstrin homology domain containing, family H (with MyTH4 domain) member 3	CCDS11434.1	chr17_38082019-38082024__TCCCCG	NA	Insertion
Subependymoma	1022013T	PLEKHM2	pleckstrin homology domain containing, family M (with RUN domain) member 2	NM_015164	chr1_15926269-15926269_C_T	372P>L	Substitution
Subependymoma	1022013T	PMP22	peripheral myelin protein 22	CCDS11168.1	chr17_15075101-15075101_G_A	114A>V	Substitution
Subependymoma	1022013T	POM121-2	POM121 transmembrane nucleoporin C	ENST00000257665	chr7_74909685-74909685_A_G	82F>S	Substitution
Subependymoma	1022013T	PPP1R12C	protein phosphatase 1, regulatory subunit 12C	CCDS12916.1	chr19_60320467-60320467_G_A	86A>V	Substitution
Subependymoma	1022013T	PPP4R4	protein phosphatase 4, regulatory subunit 4	CCDS9921.1	chr14_93744563-93744563_A_C	67Q>H	Substitution
Subependymoma	1022013T	PRDM13	PR domain containing 13	CCDS43487.1	chr6_100168346-100168346__CCG	372->P	Insertion
Subependymoma	1022013T	PRDM2	PR domain containing 2, with ZNF domain	ENST00000400803	chr1_13981972-13981972_G_A	NA	Substitution
Subependymoma	1022013T	PRKCSH	protein kinase C substrate 80K-H	CCDS32911.1	chr19_11419370-11419372_GGA_	NA	Deletion
Subependymoma	1022013T	PRKD1	protein kinase D1	CCDS9637.1	chr14_29466383-29466388__GGACCC	NA	Insertion
Subependymoma	1022013T	PRRC2A	proline-rich coiled-coil 2A	CCDS4708.1	chr6_31703763-31703774_ACCTGCTGCCCC	NA	Deletion
Subependymoma	1022013T	PRSS23	protease, serine, 23	CCDS8278.1	chr11_86196829-86196829_G_T	166A>S	Substitution
Subependymoma	SBP 8292014T	PRSS35	protease, serine, 35	CCDS4999.1	chr6_84290559-84290559_C_T	227A>V	Substitution
Subependymoma	1022013T	PRSS57	protease, serine, 57	CCDS12041.1	chr19_636777-636777_C_T	264R>Q	Substitution
Subependymoma	8065T	PTGIS	prostaglandin I2 (prostacyclin) synthase	CCDS13419.1	chr20_47589564-47589564_C_T	208R>H	Substitution
Subependymoma	1022013T	PTH2R	parathyroid hormone 2 receptor	CCDS2383.1	chr2_209066312-209066312_C_T	446R>C	Substitution
Subependymoma	1022013T	PTPN1	protein tyrosine phosphatase, non-receptor type 1	CCDS13430.1	chr20_48624559-48624563_ATATT_	NA	Deletion
Subependymoma	1022013T	PTPRC	protein tyrosine phosphatase, receptor type, C	CCDS1397.1	chr1_196991682-196991682_G_A	1222V>I	Substitution
Subependymoma	1022013T	PYGB	phosphorylase, glycogen; brain	CCDS13171.1	chr20_25176984-25176984_C_G	57A>G	Substitution
Subependymoma	1022013T	QPCTL	glutaminyl-peptide cyclotransferase-like	CCDS12672.1	chr19_50887883-50887883_C_T	28R>C	Substitution
Subependymoma	1022013T	RASAL1	RAS protein activator like 1 (GAP1 like)	CCDS9165.1	chr12_112037844-112037844_G_A	328R>W	Substitution
Subependymoma	1022013T	RCC1	regulator of chromosome condensation 1	CCDS41295.1	chr1_28735016-28735016_C_G	267S>R	Substitution
Subependymoma	SBP 8292014T	RHCG	Rh family, C glycoprotein	CCDS10351.1	chr15_87822071-87822071_C_T	NA	Substitution
Subependymoma	1022013T	RRM2B	ribonucleotide reductase M2 B (TP53 inducible)	NM_001172477	chr8_103320016-103320016__G	NA	Insertion
Subependymoma	1022013T	RTKN2	rhotekin 2	CCDS7263.1	chr10_63646920-63646925_GCTTCA_	NA	Deletion
Subependymoma	1022013T	RTL1	retrotransposon-like 1	NM_001134888	chr14_100420424-100420424__TCT	152E>EK	Insertion
Subependymoma	SBP 8292014T	RYR2	ryanodine receptor 2 (cardiac)	NM_001035	chr1_235778463-235778463_G_A	1006V>M	Substitution
Subependymoma	8065T	RYR3	ryanodine receptor 3	NM_001036	chr15_31817976-31817976_T_C	2517Y>H	Substitution
Subependymoma	1022013T	SAAL1	serum amyloid A-like 1	CCDS31439.1	chr11_18084135-18084135__CGG	10P>PR	Insertion
Subependymoma	1022013T	SCARF2	scavenger receptor class F, member 2	CCDS13779.1	chr22_19109769-19109769_C_G	837A>P	Substitution
Subependymoma	1022013T	SENP6	SUMO1/sentrin specific peptidase 6	CCDS43483.1	chr6_76443657-76443657_G_A	598G>R	Substitution
Subependymoma	1022013T	SERAC1	serine active site containing 1	CCDS5255.1	chr6_158454514-158454514_T_G	595I>L	Substitution
Subependymoma	1022013T	SERPINC1	serpin peptidase inhibitor, clade C (antithrombin), member 1	CCDS1313.1	chr1_172139799-172139799_C_A	416A>S	Substitution
Subependymoma	1022013T	SFRP4	secreted frizzled-related protein 4	CCDS5453.1	chr7_37915796-37915796_A_G	268L>P	Substitution
Subependymoma	1022013T	SGCD	sarcoglycan, delta (35kDa dystrophin-associated glycoprotein)	NM_000337	chr5_155704088-155704088_G_C	5E>D	Substitution
Subependymoma	1022013T	SH3RF3	SH3 domain containing ring finger 3	NM_001099289	chr2_109432081-109432081_C_G	618Q>E	Substitution
Subependymoma	1022013T	SIGLEC5	sialic acid binding Ig-like lectin 5	CCDS33088.1	chr19_56823069-56823069__G	NA	Insertion
Subependymoma	1022013T	SIRT6	sirtuin 6	CCDS12122.1	chr19_4125758-4125758_G_C	308N>K	Substitution
Subependymoma	1022013T	SIVA1	SIVA1, apoptosis-inducing factor	CCDS9992.1	chr14_104290626-104290626_C_G	15Q>E	Substitution
Subependymoma	1022013T	SLC19A3	solute carrier family 19 (thiamine transporter), member 3	CCDS2468.1	chr2_228272054-228272054_T_C	207I>M	Substitution
Subependymoma	1022013T	SLC27A2	solute carrier family 27 (fatty acid transporter), member 2	CCDS10133.1	chr15_48277034-48277034_A_G	175D>G	Substitution
Subependymoma	1022013T	SLC30A2	solute carrier family 30 (zinc transporter), member 2	CCDS30644.1	chr1_26241695-26241695_T_C	222I>V	Substitution
Subependymoma	8065T	SLC4A5	solute carrier family 4 (sodium bicarbonate cotransporter), member 5	CCDS1936.1	chr2_74385128-74385128_C_G	89R>S	Substitution
Subependymoma	1022013T	SMYD3	SET and MYND domain containing 3	CCDS31083.1	chr1_244157871-244157871_G_C	170I>M	Substitution
Subependymoma	1022013T	SNAP91	synaptosomal-associated protein, 91kDa	NM_001242792	chr6_84327343-84327343_C_T	829G>R	Substitution
Subependymoma	1022013T	SORL1	sortilin-related receptor, L(DLR class) A repeats containing	CCDS8436.1	chr11_120971589-120971589_C_T	1473R>X	Substitution
Subependymoma	1022013T	SPATA33	spermatogenesis associated 33	CCDS10983.1	chr16_88263393-88263393__TG	NA	Insertion
Subependymoma	1022013T	SPRED1	sprouty-related, EVH1 domain containing 1	CCDS32193.1	chr15_36401806-36401806_A_T	94I>F	Substitution
Subependymoma	1022013T	SRRM5	serine/arginine repetitive matrix 5	NM_001145641	chr19_48808548-48808548_A_G	145I>M	Substitution
Subependymoma	1022013T	SSPO	SCO-spondin	NM_198455	chr7_149152524-149152524_G_A	4557R>Q	Substitution

Subependymoma	1022013T	ST14	suppression of tumorigenicity 14 (colon carcinoma)	CCDS8487.1	chr11_129583751-129583751_C_T	744A>V	Substitution
Subependymoma	1022013T	STARD9	StAR-related lipid transfer (START) domain containing 9	NM_020759	chr15_40764741-40764741_G_A	1225G>S	Substitution
Subependymoma	1022013T	STK3	serine/threonine kinase 3	NM_001256312	chr8_99629370-99629370_T_C	NA	Substitution
Subependymoma	1022013T	SYNPO2	synaptopodin 2	CCDS34054.1	chr4_120171338-120171338_C_G	654P>A	Substitution
Subependymoma	1022013T	SYT6	synaptotagmin VI	CCDS871.1	chr1_114481800-114481800_T_C	219Y>C	Substitution
Subependymoma	1022013T	TACC2	transforming, acidic coiled-coil containing protein 2	CCDS7626.1	chr10_123836590-123836590_G_A	1529E>K	Substitution
Subependymoma	1022013T	TAOK2	TAO kinase 2	CCDS10663.1	chr16_29904265-29904265_G_C	551E>D	Substitution
Subependymoma	1022013T	TAS1R2	taste receptor, type 1, member 2	CCDS187.1	chr1_19053866-19053866_G_A	229R>W	Substitution
Subependymoma	9521T	TBC1D9B	TBC1 domain family, member 9B (with GRAM domain)	CCDS43408.1	chr5_179264410-179264410_C_T	43V>M	Substitution
Subependymoma	1022013T	TBX3	T-box 3	CCDS9176.1	chr12_113594390-113594390_T_C	624H>R	Substitution
Subependymoma	1022013T	TCIRG1	T-cell, immune regulator 1, ATPase, H+ transporting, lysosomal V0 subunit A3	CCDS8177.1	chr11_67567476-67567476_C_T	189A>V	Substitution
Subependymoma	1022013T	TCRBV12S2A1T	UNVERIFIED: T cell receptor beta variable 10-1(gene/pseudogene)	ENST00000390364	chr7_141768792-141768792_G_T	98E>X	Substitution
Subependymoma	1022013T	TCRBV6S6A2T	T cell receptor beta variable 7-7	ENST00000390377	chr7_141880496-141880496_G_A	83R>Q	Substitution
Subependymoma	1022013T	TEX9	testis expressed 9	CCDS10157.1	chr15_54445088-54445088_C_A	16T>N	Substitution
Subependymoma	1022013T	THSD7A	thrombospondin, type I, domain containing 7A	NM_015204	chr7_11480547-11480547_C_T	731G>S	Substitution
Subependymoma	1022013T	TIMM44	translocase of inner mitochondrial membrane 44 homolog (yeast)	CCDS12192.1	chr19_7903576-7903576_G_T	308P>Q	Substitution
Subependymoma	1022013T	TMEM168	transmembrane protein 168	CCDS5757.1	chr7_112202463-112202463_G_C	NA	Substitution
Subependymoma	1022013T	TMEM184A	transmembrane protein 184A	CCDS43537.1	chr7_1553180-1553180__GCC	392G>GA	Insertion
Subependymoma	1022013T	TMEM37	transmembrane protein 37	CCDS33281.1	chr2_119911122-119911127__GTGTGC	NA	Insertion
Subependymoma	1022013T	TMEM80	transmembrane protein 80	NM_001276274	chr11_694605-694605_A_	NA	Deletion
Subependymoma	1022013T	TMPO	thymopoietin	CCDS31879.1	chr12_97464360-97464360__T	NA	Insertion
Subependymoma	1022013T	TMPRSS11A	transmembrane protease, serine 11A	CCDS3519.1	chr4_68480360-68480360_A_C	92F>C	Substitution
Subependymoma	1022013T	TMPRSS15	transmembrane protease, serine 15	CCDS13571.1	chr21_18623377-18623377_T_C	587E>G	Substitution
Subependymoma	1022013T	TMPRSS9	transmembrane protease, serine 9	CCDS12088.1	chr19_2354185-2354185_C_T	187A>V	Substitution
Subependymoma	1022013T	TOR1A	torsin family 1, member A (torsin A)	CCDS6930.1	chr9_131620977-131620977_G_A	163A>V	Substitution
Subependymoma	1022013T	TRAPPC12	trafficking protein particle complex 12	CCDS1652.1	chr2_3370926-3370926_C_G	175F>L	Substitution
Subependymoma	1022013T	TRMT44	tRNA methyltransferase 44 homolog (S. cerevisiae)	CCDS3402.1	chr4_8528613-8528615_GAA_	361RK>R	Deletion
Subependymoma	1022013T	TRPM1	transient receptor potential cation channel, subfamily M, member 1	CCDS10024.2	chr15_29115105-29115105_G_A	857P>L	Substitution
Subependymoma	1022013T	TRPM3	transient receptor potential cation channel, subfamily M, member 3	CCDS43835.1	chr9_72647866-72647866__A	NA	Insertion
Subependymoma	1022013T	TSLP	thymic stromal lymphopoietin	CCDS4101.1	chr5_110435557-110435557_G_A	24G>R	Substitution
Subependymoma	1022013T	TTLL11	tubulin tyrosine ligase-like family, member 11	CCDS6834.2	chr9_123895158-123895163__TGGCCT	NA	Insertion
Subependymoma	1022013T	TUSC1	tumor suppressor candidate 1	CCDS34999.1	chr9_25668178-25668178__CGC	48->A	Insertion
Subependymoma	1022013T	UFSP2	UFM1-specific peptidase 2	CCDS3842.1	chr4_186573499-186573499_T_G	NA	Substitution
Subependymoma	1022013T	UPK1A	uroplakin 1A	CCDS12470.1	chr19_40858717-40858717_G_A	202E>K	Substitution
Subependymoma	1022013T	USH2A	Usher syndrome 2A (autosomal recessive, mild)	CCDS31025.1	chr1_214006685-214006685_C_T	3670E>K	Substitution
Subependymoma	1022013T	USH2A	Usher syndrome 2A (autosomal recessive, mild)	CCDS31025.1	chr1_214233077-214233077_T_G	2238E>A	Substitution
Subependymoma	1022013T	USP40	ubiquitin specific peptidase 40	ENST00000251722	chr2_234094479-234094479__TTC	672K>RK	Insertion
Subependymoma	1022013T	USP42	ubiquitin specific peptidase 42	ENST00000404835	chr7_6160090-6160090_A_G	793M>V	Substitution
Subependymoma	1022013T	USP43	ubiquitin specific peptidase 43	NM_153210	chr17_9520909-9520909_G_A	319V>I	Substitution
Subependymoma	9521T	VWCE	von Willebrand factor C and EGF domains	CCDS8002.1	chr11 60815562-60815562 G A	58A>V	Substitution
Subependymoma	1022013T	VWF	von Willebrand factor	CCDS8539.1	chr12_5932969-5932969_G_A	2647T>M	Substitution
Subependymoma	1022013T	WDR65	WD repeat domain 65	NM_001195831	chr1_43461221-43461221_G_A	891R>Q	Substitution
Subependymoma	8065T	WDR86	WD repeat domain 86	NM 001284260	chr7 150724103-150724103 C T	140V>M	Substitution
Subependymoma	1022013T	ZAN	zonadhesin (gene/pseudogene)	ENST00000349350	chr7_100190950-100190952_ACA_	1097YN>Y	Deletion
Subependymoma	1022013T	ZC3H7B	zinc finger CCCH-type containing 7B	CCDS14013.1	chr22_40064996-40064996_C_T	224T>M	Substitution
Subependymoma	1022013T	ZCCHC6	zinc finger, CCHC domain containing 6	CCDS35057.1	chr9_88128633-88128633_T_C	618I>V	Substitution
Subependymoma	1022013T	ZNF211	zinc finger protein 211	NM_001265597	chr19_62843151-62843151_G_	NA	Deletion
Subependymoma	1022013T	ZNF275	zinc finger protein 275	ENST00000095634	chrX_152263422-152263430_ACCTCTGTG_	NA	Deletion
Subependymoma	1022013T	ZNF408	zinc finger protein 408	CCDS7923.1	chr11_46681298-46681309_TGACAGAAGTGG_	NA	Deletion
Subependymoma	1022013T	ZNF541	zinc finger protein 541	NM_001277075	chr19_52740208-52740208_G_A	464P>S	Substitution
Subependymoma	1022013T	ZNF668	zinc finger protein 668	CCDS10701.1	chr16_30981106-30981106_C_A	NA	Substitution
Subependymoma	1022013T	ZNF717	zinc finger protein 717	NM_001128223	chr3_75870519-75870519_C_G	315W>C	Substitution
Subependymoma	1022013T	ZNF747	zinc finger protein 747	CCDS10682.1	chr16_30453053-30453053_C_T	150R>Q	Substitution
Subependymoma	1022013T	ZNFX1	zinc finger, NFX1-type containing 1	CCDS13417.1	chr20_47321219-47321219_C_T	179M>I	Substitution

Supplementary Table 1C. Somatic Mutations in Gangliogliomas

Tumor	Sample ID	Gene	Gene Description	Gene Accession	Nucleotide	Amino Acid	Mutation Type
Ganglioglioma	7933T	APOF	apolipoprotein F	CCDS44923.1	chr12 56755165-56755165 C A	275K>N	Substitution
Ganglioglioma	8306T	B4GALT5	UDP-Gal:betaGlcNAc beta 1,4-galactosyltransferase, polypeptide 5	CCDS13420.1	chr20_48257072-48257072_T_G	246Y>S	Substitution
Ganglioglioma	7939T	BRAF	v-raf murine sarcoma viral oncogene homolog B	CCDS5863.1	chr7 140453136-140453136 A T	600V>E	Substitution
Ganglioglioma	7933T	ERMARD	ER membrane-associated RNA degradation	CCDS34576.1	chr6 170176740-170176740 G C	570Q>H	Substitution
Ganglioglioma	7939T	FAM83A	family with sequence similarity 83, member A	CCDS6340.1	chr8_124204071-124204071_C_T	170T>M	Substitution
Ganglioglioma	7933T	FGFR1	fibroblast growth factor receptor 1	CCDS55223.1	chr8_38274851-38274851_T_C	577N>D	Substitution
Ganglioglioma	8306T	NF1	neurofibromin 1	CCDS42292.1	chr17 29683573-29683573 A T	2571R>X	Substitution
Ganglioglioma	8306T	NPEPPS	aminopeptidase puromycin sensitive	CCDS45721.1	chr17_45669359-45669359_T_G	433F>C	Substitution
Ganglioglioma	7939T	PM20D1	peptidase M20 domain containing 1	CCDS1460.1	chr1_205813958-205813958_G_T	186S>Y	Substitution
Ganglioglioma	7933T	SLC25A2	solute carrier family 25 (mitochondrial carrier; ornithine transporter) member 2	CCDS4258.1	chr5_140682837-140682837_G_A	199A>V	Substitution
Ganglioglioma	CGLI62PT1	ZDHHC11	zinc finger, DHHC-type containing 11	CCDS3857.1	chr5_837553-837553_C_G	276R>P	Substitution

Supplementary Figure 1D. Somatic mutations in Hemangioblastoma

Tumor	Sample ID	Gene	Gene Description	Gene Accession	Nucleotide	Amino Acid	Mutation Type
Hemangioblastoma	CGLI 33	EXOC7	exocyst complex component 7	CCDS32738.1	chr17_71592192-71592192_C_T	NA	Substitution
Hemangioblastoma	CGLI 33	MTPN	myotrophin	CCDS5842.1	chr7_135265270-135265270_C_T	101G>R	Substitution
Hemangioblastoma	CGLI 33	NOTCH3	Notch homolog 3 (Drosophila)	CCDS12326.1	chr19_15132720-15132720_C_T	2240S>N	Substitution
Hemangioblastoma	CGLI 33	NPEPPS	aminopeptidase puromycin sensitive	NM_006310	chr17_43024358-43024358_T_G	433F>C	Substitution
Hemangioblastoma	CGLI 33	OPRK1	opioid receptor; kappa 1	CCDS6152.1	chr8_54304740-54304740_C_A	271R>S	Substitution

Supplementary Figure 1E. Somatic Mutations in Anaplastic Oligodendroglioma

Tumor	Sample ID	Gene	Gene Description	Gene Accession	Nucleotide	Amino Acid	Mutation Type
Anaplastic Oligodendroglioma	7935T	AHNAK	AHNAK nucleoprotein	CCDS31584.1	chr11_62046355-62046355_A_G	4037I>T	Substitution
Anaplastic Oligodendroglioma	7935T	ARMCX1	armadillo repeat containing, X-linked 1	CCDS14487.1	chrX_100694731-100694731_T_	NA	Deletion
Anaplastic Oligodendroglioma	7935T	CHRD	chordin	CCDS3266.1	chr3_185585055-185585055_G_A	493A>T	Substitution
Anaplastic Oligodendroglioma	7935T	COL6A3	collagen, type VI, alpha 3	CCDS33412.1	chr2_237942455-237942455_G_A	1464R>X	Substitution
Anaplastic Oligodendroglioma	7935T	DIP2C	DIP2 disco-interacting protein 2 homolog C (Drosophila)	CCDS7054.1	chr10_522435-522435_A_C	41S>A	Substitution
Anaplastic Oligodendroglioma	7935T	FLNA	filamin A, alpha	CCDS44021.1	chrX_153236272-153236272_G_A	1732A>V	Substitution
Anaplastic Oligodendroglioma	7935T	GLI3	GLI family zinc finger 3	CCDS5465.1	chr7_41971469-41971469_C_T	1243A>T	Substitution
Anaplastic Oligodendroglioma	7935T	HBS1L	HBS1-like (S. cerevisiae)	CCDS5173.1	chr6_135360320-135360320_T_C	236K>R	Substitution
Anaplastic Oligodendroglioma	7935T	IDH1	isocitrate dehydrogenase 1 (NADP+), soluble	CCDS2381.1	chr2_208821358-208821358_G_A	132R>C	Substitution
Anaplastic Oligodendroglioma	7935T	KIAA1462	KIAA1462	CCDS41500.1	chr10_30356727-30356727_C_T	786V>M	Substitution
Anaplastic Oligodendroglioma	7935T	KMT2C	lysine (K)-specific methyltransferase 2C	CCDS5931.1	chr7_151576189-151576189_G_A	755Q>X	Substitution
Anaplastic Oligodendroglioma	7935T	LYST	lysosomal trafficking regulator	CCDS31062.1	chr1_234039599-234039599_G_T	381T>K	Substitution
Anaplastic Oligodendroglioma	7935T	MAP4	microtubule-associated protein 4	CCDS33750.1	chr3_47938277-47938277_G_A	171P>S	Substitution
Anaplastic Oligodendroglioma	7935T	MIPEP	mitochondrial intermediate peptidase	CCDS9303.1	chr13_23342156-23342156_T_C	261D>G	Substitution
Anaplastic Oligodendroglioma	7935T	NLGN3	neuroligin 3	CCDS14407.1	chrX_70306335-70306335_G_A	717R>H	Substitution
Anaplastic Oligodendroglioma	7935T	NOL12	nucleolar protein 12	CCDS13955.1	chr22_36417173-36417173_G_A	176V>I	Substitution
Anaplastic Oligodendroglioma	7935T	NUDCD2	NudC domain containing 2	CCDS4361.1	chr5_162816667-162816667_A_	NA	Deletion
Anaplastic Oligodendroglioma	7935T	OR5I1	olfactory receptor, family 5, subfamily I, member 1	CCDS7949.1	chr11_55459914-55459914__A	NA	Insertion
Anaplastic Oligodendroglioma	7935T	PDE2A	phosphodiesterase 2A, cGMP-stimulated	CCDS8216.1	chr11_71977893-71977893_A_G	305S>P	Substitution
Anaplastic Oligodendroglioma	7935T	PIK3CA	phosphatidylinositol-4,5-bisphosphate 3-kinase, catalytic subunit alpha	CCDS43171.1	chr3_180434712-180434712_A_G	1025T>A	Substitution
Anaplastic Oligodendroglioma	7935T	PRC1	protein regulator of cytokinesis 1	CCDS32334.1	chr15_89314679-89314679_A_G	511Y>H	Substitution
Anaplastic Oligodendroglioma	7935T	RBBP6	retinoblastoma binding protein 6	CCDS10621.1	chr16_24488265-24488265_A_G	918E>G	Substitution
Anaplastic Oligodendroglioma	7935T	REEP3	receptor accessory protein 3	NM_001001330	chr10_65049569-65049569_T_A	NA	Substitution
Anaplastic Oligodendroglioma	7935T	RNF26	ring finger protein 26	CCDS8419.1	chr11_118712196-118712196_A_G	385D>G	Substitution
Anaplastic Oligodendroglioma	7935T	SIM1	single-minded homolog 1 (Drosophila)	CCDS5045.1	chr6_101002848-101002848_C_T	249V>M	Substitution
Anaplastic Oligodendroglioma	7935T	SIRT7	sirtuin 7	CCDS11792.1	chr17_77463744-77463744_C_T	348R>H	Substitution
Anaplastic Oligodendroglioma	7935T	SLC10A3	solute carrier family 10, member 3	CCDS14755.1	chrX_153369359-153369359_C_T	372R>H	Substitution
Anaplastic Oligodendroglioma	7935T	SOGA1	suppressor of glucose, autophagy associated 1	NM_080627	chr20_34877595-34877595_A_G	555V>A	Substitution
Anaplastic Oligodendroglioma	7935T	TARS	threonyl-tRNA synthetase	CCDS3899.1	chr5_33496910-33496911_TC_	NA	Deletion
Anaplastic Oligodendroglioma	7935T	TPST1	tyrosylprotein sulfotransferase 1	CCDS5533.1	chr7_65343670-65343670_G_T	275A>S	Substitution
Anaplastic Oligodendroglioma	7935T	TUBE1	tubulin, epsilon 1	CCDS5100.1	chr6_112499842-112499842_A_G	409F>L	Substitution
Anaplastic Oligodendroglioma	7935T	WDR64	WD repeat domain 64	NM_144625	chr1_240005102-240005102_T_G	825S>A	Substitution
Anaplastic Oligodendroglioma	7935T	ZBTB26	zinc finger and BTB domain containing 26	CCDS6847.1	chr9_124721241-124721241_A_G	265V>A	Substitution
Anaplastic Oligodendroglioma	7935T	ZNF517	zinc finger protein 517	CCDS6434.1	chr8_146004114-146004114_C_T	337R>W	Substitution
Anaplastic Oligodendroglioma	7935T	ZNFX1	zinc finger, NFX1-type containing 1	CCDS13417.1	chr20_47320435-47320435_G_A	441R>C	Substitution
