# Supplementary material for: Genomic Landscape of Intramedullary Spinal Cord Gliomas
Source: Sci Rep. 2019 Dec 10;9:18722. doi: 10.1038/s41598-019-54286-9 (PMC6904446; doi:10.1038/s41598-019-54286-9)
Supplement: Supplementary file 2 — Supplementary Table 2 [file 41598_2019_54286_MOESM2_ESM.pdf]

Genomic Landscape of Intramedullary Spinal Cord Gliomas

Ming Zhang, Ph.D.[1,+], Rajiv R. Iyer, M.D.[2,+], Tej D. Azad M.D., M.S. [2,3,+], Qing Wang, Ph.D.[1], Tomas Garzon-Muvdi, M.D.[2,4], Joanna Wang M.D.[5], Ann Liu M.D.[2], Peter Burger M.D.[6], Charles Eberhart M.D.,PhD[6], Fausto J. Rodriguez[6], M.D., Daniel M. Sciubba M.D.[2], Jean-Paul Wolinsky M.D.[2,7] , Ziya Gokaslan M.D.[2,8], Mari Groves M.D.[2], George I. Jallo, M.D. [2,9,*], Chetan Bettegowda, M.D., Ph.D.[1,2*]

Supplementary Table 2. Clinicopathologic characteristics.

Tumor	Age at surgery (years)	Sex	Spinal location	Number of spinal levels	Follow up duration (mo)
Pilocytic Astrocytoma:					
WGS-SCA 2 PT	12	M	Thoracic	3	24
WGS-SCA 1 PT	13	F	Thoracic	6	0
CGLI 15	69	F	Cervical	2	2
9922T	11	F	Thoracic	5	11
7937T	11	M	Cervicothoracic	8	2
7739T	52	M	Thoracic	2	27
CGLI 44 PT	5	F	Cervical	3	48
Grade II Astrocytoma:					
WGS-SCA 3 PT	16	M	Thoracic	2	12
CGLI 39	56	M	Cervical	2	50
7982T	1	F	Cervicothoracic	6	0
CGLI 58PT	32	M	Thoracic	3	2
CGLI 02PT	33	F	Thoracic	1	5
Anaplastic Astrocytoma:					
8068T	1	F	Cervicothoracic	8	12
7929T	26	F	Thoracic	5	12
Glioblastoma:					
CGLI 36	11	M	Thoracolumbar	4	1
7960T	9	M	Cervical	7	1
Ependymoma					
SE44PT	35	M	Cervical	4	16
SE39PT	57	F	Cervical	2	29
SE07PT	33	F	Cervical	3	0
SE05PT	20	F	Cervical	3	0
CGLI 11	50	F	Thoracic	1	0
7924T	38	M	Cervical	3	0
SE01PT	43	F	Thoracic	2	7
8648T	61	F	Thoracic	3	7
8540T	24	M	Cervicothoracic	7	16
8527T	64	M	Cervical	2	21
8520T	55	F	Cervical	4	16
8129T	40	F	Cervical	3	34
7934T	35	M	Lumbar	1	48
7927T	26	F	Cervical	4	52
7962T	50	M	Cervicothoracic	7	0
CGLI 42PT1	31	F	Thoracic	6	36
SE53PT	64	F	Thoracic	4	45
SE52PT	50	F	Cervical	4	0
Myxopapillary Ependymoma					
CGLI 25	72	F	Lumbar	2	48
CGLI 13	27	M	Lumbar	3	3
8543T	12	F	Thoracolumbar	5	22
8495T	34	F	Lumbar	3	21

9625T	46	F	Lumbar	1	49
8014T	26	F	Lumbar	1	42
Subependymoma:					
SE42PT	38	F	Cervicothoracic	10	0
8065T	36	F	Thoracic	2	12
9521T	51	F	Thoracic	3	20
SBP 082914T	unk	unk	unk	unk	unk
1022013T	unk	unk	unk	unk	unk
Ganglioglioma:					
7933T	18	M	Thoracolumbar	3	3
8306T	20	M	Cervical	3	1
7939T	20	M	Cervical	2	3
CGLI 62PT1	unk	unk	unk	unk	unk
Hemangioblastoma:					
CGLI 33	34	M	Cervical	3	8
Anaplastic Oligodendroglioma:					
7935T	36	F	Thoracic	3	10
**Average**	33 ± 18.8			3.6 ± 2.1	16.6 ± 17.3

unk - Unknown
