# Supplementary material for: Genomic Landscape of Intramedullary Spinal Cord Gliomas
Source: Sci Rep. 2019 Dec 10;9:18722. doi: 10.1038/s41598-019-54286-9 (PMC6904446; doi:10.1038/s41598-019-54286-9)
Supplement: Supplementary file 3 — Supplementary Table 3 [file 41598_2019_54286_MOESM3_ESM.pdf]

Genomic Landscape of Intramedullary Spinal Cord Gliomas

Ming Zhang, Ph.D.[1,+], Rajiv R. Iyer, M.D.[2,+], Tej D. Azad M.D., M.S. [2,3,+], Qing Wang, Ph.D.[1], Tomas Garzon-Muvdi, M.D.[2,4], Joanna Wang M.D.[5], Ann Liu M.D.[2], Peter Burger M.D.[6], Charles Eberhart M.D.,PhD[6], Fausto J. Rodriguez[6], M.D., Daniel M. Sciubba M.D.[2], Jean-Paul Wolinsky M.D.[2,7] , Ziya Gokaslan M.D.[2,8], Mari Groves M.D.[2], George I. Jallo, M.D. [2,9,*], Chetan Bettegowda, M.D., Ph.D.[1,2*]

Supplementary Table 3. Ependymoma IPA results. Pathways and functions considered enriched if *P* < (0.05/20 = 0.0025)

	**Top canonical pathways**	***P* value**	**Overlap**	**Molecular and Cellular functions**	**p-value**	**#Molecules**
**Ependymomas**						
Classic ependymoma (18)	nNOS signaling	1.42E-02	4.9%	Cell death and survival	4.89E-02 - 1.04E-03	5
	Histidine degradation	3.48E-02	12.5%	Cell morphology	4.76E-02 - 2.74E-03	21
	Sphingomyelin metabolism	3.48E-02	12.5%	Cell cycle	4.76E-02 - 4.42E-03	9
	HIPPO signaling	5.68E-01	2.3%	Cell-to-cell signaling and interaction	4.76E-02 - 4.42E-03	11
	Calcium signaling	6.33E-02	1.5%	Cellular assembly and organization	4.76E-02 - 4.42E-03	26
Subependymoma (6)*	Gs signaling	***2.02E-04***	2.7%	Molecular transport	4.61E-02 - 6.30E-06	7
	nNOS signaling	***8.40E-04***	4.9%	Cell morphology	4.61E-02 - 6.12E-04	9
	Netrin signaling	***2.10E-03***	3.1%	Cellular function and maintenance	4.61E-02 - 6.12E-04	8
	Methionine salvage II	***3.14E-03***	33.3%	Carbohydrate metabolism	1.77E-02 - 1.01E-03	4
	Prostanoid biosynthesis	9.39E-03	11.1%	Energy production	1.01E-03 - 1.01E-03	2
Myxopapillary ependymoma (6)	Estrogen receptor signaling	1.32E-02	1.6%	Cell morphology	4.81E-02 - 5.31E-04	7

	Ketogenesis	1.36E-02	10.0%	Cellular function and maintenance	4.02E-02 - 8.20E-04	8
	Ketolysis	1.36E-02	10.0%	Cell-to-cell signaling and interaction	4.02E-02 - 1.37E-03	5
	Mevalonate pathway I	1.76E-02	7.7%	Cellular assembly and organization	4.02E-02 - 1.37E-03	7
	Isoleucine degradation I	1.90E-02	7.1%	Cellular development	4.02E-02 - 1.37E-03	6

*one specimen with 315 unique somatic mutations analyzed separately
