# Supplementary material for: Genomic Landscape of Intramedullary Spinal Cord Gliomas
Source: Sci Rep. 2019 Dec 10;9:18722. doi: 10.1038/s41598-019-54286-9 (PMC6904446; doi:10.1038/s41598-019-54286-9)
Supplement: Supplementary file 4 — Supplementary Table 4 [file 41598_2019_54286_MOESM4_ESM.pdf]

Genomic Landscape of Intramedullary Spinal Cord Gliomas

Ming Zhang, Ph.D.[1,+], Rajiv R. Iyer, M.D.[2,+], Tej D. Azad M.D., M.S. [2,3,+], Qing Wang, Ph.D.[1], Tomas Garzon-Muvdi, M.D.[2,4], Joanna Wang M.D.[5], Ann Liu M.D.[2], Peter Burger M.D.[6], Charles Eberhart M.D.,PhD[6], Fausto J. Rodriguez[6], M.D., Daniel M. Sciubba M.D.[2], Jean-Paul Wolinsky M.D.[2,7] , Ziya Gokaslan M.D.[2,8], Mari Groves M.D.[2], George I. Jallo, M.D. [2,9,*], Chetan Bettegowda, M.D., Ph.D.[1,2*]

Supplementary Table 4. Recurrent Copy Number Changes

Tumor Type	Sample ID	Gene	Gene Name	Gene Accession	Nucleotide position	Amplification	Mutation type
Ependymoma II	7692T	ABHD13	abhydrolase domain containing 13	ENSG00000139826	chr13:108870762-108886603	NA	Deletion
Ependymoma II	7934T	ABHD13	abhydrolase domain containing 13	ENSG00000139826	chr13:108870762-108886603	NA	Deletion
Ependymoma II	8129T	ABHD13	abhydrolase domain containing 13	ENSG00000139826	chr13:108870762-108886603	NA	Deletion
Ependymoma II	7692T	ANGPTL3	angiopoietin-like 3	ENSG00000132855	chr1:63063157-63071976	NA	Deletion
Ependymoma II	7934T	ANGPTL3	angiopoietin-like 3	ENSG00000132855	chr1:63063157-63071976	NA	Deletion
Ependymoma II	8129T	ANGPTL3	angiopoietin-like 3	ENSG00000132855	chr1:63063157-63071976	NA	Deletion
Ependymoma II	7692T	ARL6	ADP-ribosylation factor-like 6	ENSG00000113966	chr3:97483364-97517379	NA	Deletion
Ependymoma II	7934T	ARL6	ADP-ribosylation factor-like 6	ENSG00000113966	chr3:97483364-97517379	NA	Deletion
Ependymoma II	7692T	ATG4C	autophagy related 4C, cysteine peptidase	ENSG00000125703	chr1:63249776-63330941	NA	Deletion
Ependymoma II	7934T	ATG4C	autophagy related 4C, cysteine peptidase	ENSG00000125703	chr1:63249776-63330941	NA	Deletion
Ependymoma II	8129T	ATG4C	autophagy related 4C, cysteine peptidase	ENSG00000125703	chr1:63249776-63330941	NA	Deletion
Ependymoma II	7692T	AZI2	5-azacytidine induced 2	ENSG00000163512	chr3:28363843-28390618	NA	Deletion
Ependymoma II	7934T	AZI2	5-azacytidine induced 2	ENSG00000163512	chr3:28363843-28390618	NA	Deletion
Ependymoma II	8129T	AZI2	5-azacytidine induced 2	ENSG00000163512	chr3:28363843-28390618	NA	Deletion
Ependymoma II	7692T	C14orf39	chromosome 14 open reading frame 39	ENSG00000179008	chr14:60902673-60952764	NA	Deletion
Ependymoma II	7934T	C14orf39	chromosome 14 open reading frame 39	ENSG00000179008	chr14:60902673-60952764	NA	Deletion
Ependymoma II	8129T	C14orf39	chromosome 14 open reading frame 39	ENSG00000179008	chr14:60902673-60952764	NA	Deletion
Ependymoma II	7692T	C18orf54	chromosome 18 open reading frame 54	ENSG00000166845	chr18:51885170-51908404	NA	Deletion
Ependymoma II	7934T	C18orf54	chromosome 18 open reading frame 54	ENSG00000166845	chr18:51885170-51908404	NA	Deletion
Ependymoma II	8129T	C18orf54	chromosome 18 open reading frame 54	ENSG00000166845	chr18:51885170-51908404	NA	Deletion
Ependymoma II	7692T	C3orf14	chromosome 3 open reading frame 14	ENSG00000114405	chr3:62305395-62319320	NA	Deletion
Ependymoma II	7934T	C3orf14	chromosome 3 open reading frame 14	ENSG00000114405	chr3:62305395-62319320	NA	Deletion
Ependymoma II	8129T	C3orf14	chromosome 3 open reading frame 14	ENSG00000114405	chr3:62305395-62319320	NA	Deletion
Ependymoma II	7692T	CCDC91	coiled-coil domain containing 91	ENSG00000123106	chr12:28410132-28703099	NA	Deletion
Ependymoma II	8129T	CCDC91	coiled-coil domain containing 91	ENSG00000123106	chr12:28410132-28703099	NA	Deletion
Ependymoma II	7692T	CSN1S1	casein alpha s1	ENSG00000126545	chr4:70796798-70812288	NA	Deletion
Ependymoma II	8129T	CSN1S1	casein alpha s1	ENSG00000126545	chr4:70796798-70812288	NA	Deletion
Ependymoma II	7692T	CYLC1	cylicin, basic protein of sperm head cytoskeleton 1	ENSG00000183035	chrX:83116133-83141708	NA	Deletion
Ependymoma II	7934T	CYLC1	cylicin, basic protein of sperm head cytoskeleton 1	ENSG00000183035	chrX:83116133-83141708	NA	Deletion
Ependymoma II	8129T	CYLC1	cylicin, basic protein of sperm head cytoskeleton 1	ENSG00000183035	chrX:83116133-83141708	NA	Deletion
Ependymoma II	7692T	DEFB110	defensin, beta 110 locus	ENSG00000203970	chr6:49976850-49989694	NA	Deletion
Ependymoma II	7934T	DEFB110	defensin, beta 110 locus	ENSG00000203970	chr6:49976850-49989694	NA	Deletion
Ependymoma II	7692T	DEFB112	defensin, beta 112	ENSG00000180872	chr6:50011287-50016364	NA	Deletion
Ependymoma II	7934T	DEFB112	defensin, beta 112	ENSG00000180872	chr6:50011287-50016364	NA	Deletion
Ependymoma II	8129T	DEFB112	defensin, beta 112	ENSG00000180872	chr6:50011287-50016364	NA	Deletion
Ependymoma II	7692T	DEFB113	defensin, beta 113	ENSG00000214642	chr6:49936389-49937338	NA	Deletion
Ependymoma II	7924T	DEFB113	defensin, beta 113	ENSG00000214642	chr6:49936389-49937338	NA	Deletion
Ependymoma II	7934T	DEFB113	defensin, beta 113	ENSG00000214642	chr6:49936389-49937338	NA	Deletion
Ependymoma II	8129T	DEFB113	defensin, beta 113	ENSG00000214642	chr6:49936389-49937338	NA	Deletion
Ependymoma II	7692T	DEFB114	defensin, beta 114	ENSG00000177684	chr6:49928004-49931818	NA	Deletion
Ependymoma II	7934T	DEFB114	defensin, beta 114	ENSG00000177684	chr6:49928004-49931818	NA	Deletion
Ependymoma II	8129T	DEFB114	defensin, beta 114	ENSG00000177684	chr6:49928004-49931818	NA	Deletion
Ependymoma II	7692T	DEFB133	defensin, beta 133	ENSG00000214643	chr6:49913813-49917157	NA	Deletion
Ependymoma II	7934T	DEFB133	defensin, beta 133	ENSG00000214643	chr6:49913813-49917157	NA	Deletion
Ependymoma II	7692T	DNAJB9	DnaJ (Hsp40) homolog, subfamily B, member 9	ENSG00000128590	chr7:108210188-108215294	NA	Deletion
Ependymoma II	8129T	DNAJB9	DnaJ (Hsp40) homolog, subfamily B, member 9	ENSG00000128590	chr7:108210188-108215294	NA	Deletion
Ependymoma II	7692T	ENPP4	ectonucleotide pyrophosphatase/phosphodiesterase 4 (putative)	ENSG00000001561	chr6:46097700-46114436	NA	Deletion
Ependymoma II	7934T	ENPP4	ectonucleotide pyrophosphatase/phosphodiesterase 4 (putative)	ENSG00000001561	chr6:46097700-46114436	NA	Deletion
Ependymoma II	7692T	FABP2	fatty acid binding protein 2, intestinal	ENSG00000145384	chr4:120238404-120243316	NA	Deletion
Ependymoma II	8129T	FABP2	fatty acid binding protein 2, intestinal	ENSG00000145384	chr4:120238404-120243316	NA	Deletion
Ependymoma II	7692T	FAM111B	family with sequence similarity 111, member B	ENSG00000189057	chr11:58874657-58894888	NA	Deletion
Ependymoma II	7934T	FAM111B	family with sequence similarity 111, member B	ENSG00000189057	chr11:58874657-58894888	NA	Deletion
Ependymoma II	8129T	FAM111B	family with sequence similarity 111, member B	ENSG00000189057	chr11:58874657-58894888	NA	Deletion
Ependymoma II	7692T	FDCSP	follicular dendritic cell secreted protein	ENSG00000181617	chr4:71091787-71100968	NA	Deletion
Ependymoma II	8129T	FDCSP	follicular dendritic cell secreted protein	ENSG00000181617	chr4:71091787-71100968	NA	Deletion
Ependymoma II	7692T	FGF7	fibroblast growth factor 7	ENSG00000140285	chr15:49715374-49779523	NA	Deletion
Ependymoma II	7934T	FGF7	fibroblast growth factor 7	ENSG00000140285	chr15:49715374-49779523	NA	Deletion
Ependymoma II	7692T	FSIP2	fibrous sheath interacting protein 2	ENSG00000188738	chr2:186603354-186698016	NA	Deletion
Ependymoma II	7934T	FSIP2	fibrous sheath interacting protein 2	ENSG00000188738	chr2:186603354-186698016	NA	Deletion
Ependymoma II	8129T	FSIP2	fibrous sheath interacting protein 2	ENSG00000188738	chr2:186603354-186698016	NA	Deletion
Ependymoma II	7692T	GNPDA2	glucosamine-6-phosphate deaminase 2	ENSG00000163281	chr4:44703811-44728651	NA	Deletion
Ependymoma II	7934T	GNPDA2	glucosamine-6-phosphate deaminase 2	ENSG00000163281	chr4:44703811-44728651	NA	Deletion
Ependymoma II	8129T	GNPDA2	glucosamine-6-phosphate deaminase 2	ENSG00000163281	chr4:44703811-44728651	NA	Deletion
Ependymoma II	7692T	GNPNAT1	glucosamine-phosphate N-acetyltransferase 1	ENSG00000100522	chr14:53241910-53258386	NA	Deletion
Ependymoma II	8129T	GNPNAT1	glucosamine-phosphate N-acetyltransferase 1	ENSG00000100522	chr14:53241910-53258386	NA	Deletion
Ependymoma II	7692T	GPR22	G protein-coupled receptor 22	ENSG00000172209	chr7:107110501-107116125	NA	Deletion
Ependymoma II	7934T	GPR22	G protein-coupled receptor 22	ENSG00000172209	chr7:107110501-107116125	NA	Deletion
Ependymoma II	8129T	GPR22	G protein-coupled receptor 22	ENSG00000172209	chr7:107110501-107116125	NA	Deletion
Ependymoma II	7692T	HTN1	histatin 1	ENSG00000126550	chr4:70916158-70924559	NA	Deletion
Ependymoma II	8129T	HTN1	histatin 1	ENSG00000126550	chr4:70916158-70924559	NA	Deletion
Ependymoma II	7692T	HTN3	histatin 3	ENSG00000205649	chr4:70894129-70902255	NA	Deletion
Ependymoma II	8129T	HTN3	histatin 3	ENSG00000205649	chr4:70894129-70902255	NA	Deletion
Ependymoma II	7692T	IL2	interleukin 2	ENSG00000109471	chr4:123372625-123377650	NA	Deletion
Ependymoma II	7934T	IL2	interleukin 2	ENSG00000109471	chr4:123372625-123377650	NA	Deletion
Ependymoma II	8129T	IL2	interleukin 2	ENSG00000109471	chr4:123372625-123377650	NA	Deletion
Ependymoma II	7692T	KERA	keratocan	ENSG00000139330	chr12:91444270-91452131	NA	Deletion
Ependymoma II	7934T	KERA	keratocan	ENSG00000139330	chr12:91444270-91452131	NA	Deletion
Ependymoma II	8129T	KERA	keratocan	ENSG00000139330	chr12:91444270-91452131	NA	Deletion
Ependymoma II	7692T	KRTAP22-2	keratin associated protein 22-2	ENSG00000206106	chr21:31962423-31962716	NA	Deletion
Ependymoma II	7934T	KRTAP22-2	keratin associated protein 22-2	ENSG00000206106	chr21:31962423-31962716	NA	Deletion
Ependymoma II	8129T	KRTAP22-2	keratin associated protein 22-2	ENSG00000206106	chr21:31962423-31962716	NA	Deletion
Ependymoma II	7692T	LIPJ	lipase, family member J	ENSG00000204022	chr10:90346518-90366733	NA	Deletion
Ependymoma II	7934T	LIPJ	lipase, family member J	ENSG00000204022	chr10:90346518-90366733	NA	Deletion
Ependymoma II	7692T	LRRIQ3	leucine-rich repeats and IQ motif containing 3	ENSG00000162620	chr1:74491701-74663871	NA	Deletion
Ependymoma II	7934T	LRRIQ3	leucine-rich repeats and IQ motif containing 3	ENSG00000162620	chr1:74491701-74663871	NA	Deletion
Ependymoma II	8129T	LRRIQ3	leucine-rich repeats and IQ motif containing 3	ENSG00000162620	chr1:74491701-74663871	NA	Deletion
Ependymoma II	7692T	MGAT4C	mannosyl (alpha-1,3-)-glycoprotein beta-1,4-N-acetylglucosaminyltransferase, isozyme C (putative)	ENSG00000182050	chr12:86373036-87232681	NA	Deletion
Ependymoma II	7934T	MGAT4C	mannosyl (alpha-1,3-)-glycoprotein beta-1,4-N-acetylglucosaminyltransferase, isozyme C (putative)	ENSG00000182050	chr12:86373036-87232681	NA	Deletion
Ependymoma II	8129T	MGAT4C	mannosyl (alpha-1,3-)-glycoprotein beta-1,4-N-acetylglucosaminyltransferase, isozyme C (putative)	ENSG00000182050	chr12:86373036-87232681	NA	Deletion
Ependymoma II	7692T	MSTN	myostatin	ENSG00000138379	chr2:190920425-190927455	NA	Deletion
Ependymoma II	7934T	MSTN	myostatin	ENSG00000138379	chr2:190920425-190927455	NA	Deletion
Ependymoma II	7692T	NUF2	NUF2, NDC80 kinetochore complex component	ENSG00000143228	chr1:163291722-163325553	NA	Deletion
Ependymoma II	7934T	NUF2	NUF2, NDC80 kinetochore complex component	ENSG00000143228	chr1:163291722-163325553	NA	Deletion
Ependymoma II	8129T	NUF2	NUF2, NDC80 kinetochore complex component	ENSG00000143228	chr1:163291722-163325553	NA	Deletion
Ependymoma II	7692T	OR5H1	olfactory receptor, family 5, subfamily H, member 1	ENSG00000231192	chr3:97851541-97852483	NA	Deletion

Ependymoma II	7934T	OR5H1	olfactory receptor, family 5, subfamily H, member 1	ENSG00000231192	chr3:97851541-97852483	NA	Deletion
Ependymoma II	8129T	OR5H1	olfactory receptor, family 5, subfamily H, member 1	ENSG00000231192	chr3:97851541-97852483	NA	Deletion
Ependymoma II	7692T	OR5H15	olfactory receptor, family 5, subfamily H, member 15	ENSG00000233412	chr3:97887543-97888485	NA	Deletion
Ependymoma II	7934T	OR5H15	olfactory receptor, family 5, subfamily H, member 15	ENSG00000233412	chr3:97887543-97888485	NA	Deletion
Ependymoma II	7692T	OR5T3	olfactory receptor, family 5, subfamily T, member 3	ENSG00000172489	chr11:56019675-56020698	NA	Deletion
Ependymoma II	7934T	OR5T3	olfactory receptor, family 5, subfamily T, member 3	ENSG00000172489	chr11:56019675-56020698	NA	Deletion
Ependymoma II	8129T	OR5T3	olfactory receptor, family 5, subfamily T, member 3	ENSG00000172489	chr11:56019675-56020698	NA	Deletion
Ependymoma II	7692T	ORC4	origin recognition complex, subunit 4	ENSG00000115947	chr2:148687965-148779173	NA	Deletion
Ependymoma II	7934T	ORC4	origin recognition complex, subunit 4	ENSG00000115947	chr2:148687965-148779173	NA	Deletion
Ependymoma II	8129T	ORC4	origin recognition complex, subunit 4	ENSG00000115947	chr2:148687965-148779173	NA	Deletion
Ependymoma II	7692T	PMCH	pro-melanin-concentrating hormone	ENSG00000183395	chr12:102590236-102591614	NA	Deletion
Ependymoma II	7924T	PMCH	pro-melanin-concentrating hormone	ENSG00000183395	chr12:102590236-102591614	NA	Deletion
Ependymoma II	7934T	PMCH	pro-melanin-concentrating hormone	ENSG00000183395	chr12:102590236-102591614	NA	Deletion
Ependymoma II	8129T	PMCH	pro-melanin-concentrating hormone	ENSG00000183395	chr12:102590236-102591614	NA	Deletion
Ependymoma II	7692T	SAMD9	sterile alpha motif domain containing 9	ENSG00000205413	chr7:92728825-92747336	NA	Deletion
Ependymoma II	7934T	SAMD9	sterile alpha motif domain containing 9	ENSG00000205413	chr7:92728825-92747336	NA	Deletion
Ependymoma II	8129T	SAMD9	sterile alpha motif domain containing 9	ENSG00000205413	chr7:92728825-92747336	NA	Deletion
Ependymoma II	7692T	SGOL2	shugoshin-like 2 (S. pombe)	ENSG00000163535	chr2:201390864-201448818	NA	Deletion
Ependymoma II	7934T	SGOL2	shugoshin-like 2 (S. pombe)	ENSG00000163535	chr2:201390864-201448818	NA	Deletion
Ependymoma II	8129T	SGOL2	shugoshin-like 2 (S. pombe)	ENSG00000163535	chr2:201390864-201448818	NA	Deletion
Ependymoma II	7692T	SLCO1B7	solute carrier organic anion transporter family, member 1B7 (non-functional)	ENSG00000205754	chr12:21168629-21243040	NA	Deletion
Ependymoma II	7934T	SLCO1B7	solute carrier organic anion transporter family, member 1B7 (non-functional)	ENSG00000205754	chr12:21168629-21243040	NA	Deletion
Ependymoma II	8129T	SLCO1B7	solute carrier organic anion transporter family, member 1B7 (non-functional)	ENSG00000205754	chr12:21168629-21243040	NA	Deletion
Ependymoma II	7692T	SNX16	sorting nexin 16	ENSG00000104497	chr8:82711817-82754521	NA	Deletion
Ependymoma II	8129T	SNX16	sorting nexin 16	ENSG00000104497	chr8:82711817-82754521	NA	Deletion
Ependymoma II	7692T	STATH	statherin	ENSG00000126549	chr4:70861647-70868173	NA	Deletion
Ependymoma II	8129T	STATH	statherin	ENSG00000126549	chr4:70861647-70868173	NA	Deletion
Ependymoma II	7692T	SULT1B1	sulfotransferase family, cytosolic, 1B, member 1	ENSG00000173597	chr4:70592685-70626430	NA	Deletion
Ependymoma II	8129T	SULT1B1	sulfotransferase family, cytosolic, 1B, member 1	ENSG00000173597	chr4:70592685-70626430	NA	Deletion
Ependymoma II	7692T	SULT1E1	sulfotransferase family 1E, estrogen-preferring, member 1	ENSG00000109193	chr4:70706929-70725870	NA	Deletion
Ependymoma II	8129T	SULT1E1	sulfotransferase family 1E, estrogen-preferring, member 1	ENSG00000109193	chr4:70706929-70725870	NA	Deletion
Ependymoma II	7692T	SYCP2	synaptonemal complex protein 2	ENSG00000196074	chr20:58438617-58507209	NA	Deletion
Ependymoma II	7934T	SYCP2	synaptonemal complex protein 2	ENSG00000196074	chr20:58438617-58507209	NA	Deletion
Ependymoma II	8129T	SYCP2	synaptonemal complex protein 2	ENSG00000196074	chr20:58438617-58507209	NA	Deletion
Ependymoma II	7692T	TAS2R10	taste receptor, type 2, member 10	ENSG00000121318	chr12:10977944-10978868	NA	Deletion
Ependymoma II	7934T	TAS2R10	taste receptor, type 2, member 10	ENSG00000121318	chr12:10977944-10978868	NA	Deletion
Ependymoma II	8129T	TAS2R10	taste receptor, type 2, member 10	ENSG00000121318	chr12:10977944-10978868	NA	Deletion
Ependymoma II	7692T	TAS2R13	taste receptor, type 2, member 13	ENSG00000212128	chr12:11060524-11062161	NA	Deletion
Ependymoma II	8129T	TAS2R13	taste receptor, type 2, member 13	ENSG00000212128	chr12:11060524-11062161	NA	Deletion
Ependymoma II	7692T	TC2N	tandem C2 domains, nuclear	ENSG00000165929	chr14:92246266-92333880	NA	Deletion
Ependymoma II	7934T	TC2N	tandem C2 domains, nuclear	ENSG00000165929	chr14:92246266-92333880	NA	Deletion
Ependymoma II	8129T	TC2N	tandem C2 domains, nuclear	ENSG00000165929	chr14:92246266-92333880	NA	Deletion
Ependymoma II	7692T	TFPI	tissue factor pathway inhibitor (lipoprotein-associated coagulation inhibitor)	ENSG00000003436	chr2:188328957-188419219	NA	Deletion
Ependymoma II	8129T	TFPI	tissue factor pathway inhibitor (lipoprotein-associated coagulation inhibitor)	ENSG00000003436	chr2:188328957-188419219	NA	Deletion
Ependymoma II	7692T	TNFSF18	tumor necrosis factor (ligand) superfamily, member 18	ENSG00000120337	chr1:173010359-173020103	NA	Deletion
Ependymoma II	7934T	TNFSF18	tumor necrosis factor (ligand) superfamily, member 18	ENSG00000120337	chr1:173010359-173020103	NA	Deletion
Ependymoma II	7692T	TRPC5OS	TRPC5 opposite strand	ENSG00000204025	chrX:111119278-111147213	NA	Deletion
Ependymoma II	7934T	TRPC5OS	TRPC5 opposite strand	ENSG00000204025	chrX:111119278-111147213	NA	Deletion
Ependymoma II	7692T	TTK	TTK protein kinase	ENSG00000112742	chr6:80714321-80752244	NA	Deletion
Ependymoma II	7934T	TTK	TTK protein kinase	ENSG00000112742	chr6:80714321-80752244	NA	Deletion
Ependymoma II	8129T	TTK	TTK protein kinase	ENSG00000112742	chr6:80714321-80752244	NA	Deletion
Ependymoma II	7692T	XKR9	XK, Kell blood group complex subunit-related family, member 9	ENSG00000221947	chr8:71581599-71648177	NA	Deletion
Ependymoma II	8129T	XKR9	XK, Kell blood group complex subunit-related family, member 9	ENSG00000221947	chr8:71581599-71648177	NA	Deletion
Ependymoma II	7692T	XRCC4	X-ray repair complementing defective repair in Chinese hamster cells 4	ENSG00000152422	chr5:82373316-82649579	NA	Deletion
Ependymoma II	8129T	XRCC4	X-ray repair complementing defective repair in Chinese hamster cells 4	ENSG00000152422	chr5:82373316-82649579	NA	Deletion
Ependymoma II	7692T	ZFAND6	zinc finger, AN1-type domain 6	ENSG00000086666	chr15:80351909-80430735	NA	Deletion
Ependymoma II	7934T	ZFAND6	zinc finger, AN1-type domain 6	ENSG00000086666	chr15:80351909-80430735	NA	Deletion
Ependymoma II	7934T	ZNF117	zinc finger protein 117	ENSG00000152926	chr7:64434829-64451414	NA	Deletion
Ependymoma II	8129T	ZNF117	zinc finger protein 117	ENSG00000152926	chr7:64434829-64451414	NA	Deletion
Ependymoma II	7692T	ZNF404	zinc finger protein 404	ENSG00000176222	chr19:44376514-44388116	NA	Deletion
Ependymoma II	7934T	ZNF404	zinc finger protein 404	ENSG00000176222	chr19:44376514-44388116	NA	Deletion
Ependymoma II	7692T	ZNF518A	zinc finger protein 518A	ENSG00000177853	chr10:97889471-97923517	NA	Deletion
Ependymoma II	7934T	ZNF518A	zinc finger protein 518A	ENSG00000177853	chr10:97889471-97923517	NA	Deletion
Ependymoma II	8129T	ZNF518A	zinc finger protein 518A	ENSG00000177853	chr10:97889471-97923517	NA	Deletion
Ependymoma II	7692T	ZNF644	zinc finger protein 644	ENSG00000122482	chr1:91380856-91487812	NA	Deletion
Ependymoma II	7934T	ZNF644	zinc finger protein 644	ENSG00000122482	chr1:91380856-91487812	NA	Deletion
Ependymoma II	7692T	ZNF654	zinc finger protein 654	ENSG00000175105	chr3:88188261-88193814	NA	Deletion
Ependymoma II	7934T	ZNF654	zinc finger protein 654	ENSG00000175105	chr3:88188261-88193814	NA	Deletion
Ependymoma II	8129T	ZNF654	zinc finger protein 654	ENSG00000175105	chr3:88188261-88193814	NA	Deletion
Ependymoma II	7692T	ZNF781	zinc finger protein 781	ENSG00000196381	chr19:38158649-38183216	NA	Deletion
Ependymoma II	8129T	ZNF781	zinc finger protein 781	ENSG00000196381	chr19:38158649-38183216	NA	Deletion
Myxopapillary I	CGLI 13	CTU1	cytosolic thiouridylase subunit 1 homolog (S. pombe)	ENSG00000142544	chr19:56292675-56303459	6.4	Amplification
Myxopapillary I	CGLI 25	CTU1	cytosolic thiouridylase subunit 1 homolog (S. pombe)	ENSG00000142544	chr19:56292675-56303459	5.0	Amplification
