# Supplementary material for: Genomic Landscape of Intramedullary Spinal Cord Gliomas
Source: Sci Rep. 2019 Dec 10;9:18722. doi: 10.1038/s41598-019-54286-9 (PMC6904446; doi:10.1038/s41598-019-54286-9)
Supplement: Supplementary file 5 — Supplementary Table 5 [file 41598_2019_54286_MOESM5_ESM.pdf]

Genomic Landscape of Intramedullary Spinal Cord Gliomas

Ming Zhang, Ph.D.[1,+], Rajiv R. Iyer, M.D.[2,+], Tej D. Azad M.D., M.S. [2,3,+], Qing Wang, Ph.D.[1], Tomas Garzon-Muvdi, M.D.[2,4], Joanna Wang M.D.[5], Ann Liu M.D.[2], Peter Burger M.D.[6], Charles Eberhart M.D.,PhD[6], Fausto J. Rodriguez[6], M.D., Daniel M. Sciubba M.D.[2], Jean-Paul Wolinsky M.D.[2,7] , Ziya Gokaslan M.D.[2,8], Mari Groves M.D.[2], George I. Jallo, M.D. [2,9,*], Chetan Bettegowda, M.D., Ph.D.[1,2*]

Supplementary Table 5. Astrocytoma IPA results. Pathways and functions considered enriched if *P* < (0.05/20 = 0.0025)

**Tumor subtype (n)**	**Top canonical pathways**	**p-value**	**Overlap**	**Molecular and Cellular functions**	**p-value**	**#Molecules**
**Astrocytomas**						
**Pilocytic** (8)	tRNA charging	***7.60E-04***	5.1%	Cell cycle	2.79E-02 - 1.05E-03	2
	Integrin signaling	2.19E-02	0.9%	Cell signaling	***1.05E-03 - 1.05E-03***	1
	Nucleotide excision repair pathway	3.61E-02	2.9%	Cell-to-cell signaling and interaction	4.81E-02 - 1.05E-03	4
	Thyroid hormone metabolism	4.41E-02	2.3%	Cellular assembly and organization	2.69E-02 - 1.05E-03	4
	Assembly of RNA polymerase II complex	5.11E-02	2.0%	Cellular development	4.41E-02 - 1.05E-03	7
**Grade II astrocytomas** (5)	MIF-mediated glucocorticoid regulation	2.83E-02	2.9%	Cellular movement	3.79E-02 - 2.20E-04	7
	Notch signaling	3.07E-02	2.6%	Cell-to-cell signaling and interaction	4.65E-02 - 3.13E-04	5
	Inhibition of matrix metalloproteases	3.15E-02	2.6%	Cell cycle	2.83E-02 - 8.20E-04	3
	MIF regulation of innate immunity	3.47E-02	2.3%	Cell morphology	3.87E-02 - 8.20E-04	5
	Phospholipases	4.97E-02	1.6%	Cellular assembly and organization	4.73E-02 - 8.20E-04	2
**Anaplastic astrocytomas** (2)	Agrin interactions at neuromuscular junction	2.57E-03	2.9%	Cell death and survival	***7.63E-03 - 8.87E-05***	4
	Caveolar-mediated endocytosis signaling	2.72E-03	2.8%	Cellular growth and proliferation	***7.63E-03 - 8.87E-05***	7
	Bladder cancer signaling	4.14E-03	2.3%	Cellular development	***7.63E-03 - 9.24E-05***	7
	Granulocyte adhesion and diapedesis	1.66E-02	1.1%	Cellular function and maintenance	***3.19E-03 - 1.03E-04***	4
	Regulation of the epithelial-mesenchymal	1.80E-02	1.1%	Cell cycle	***7.63E-03 - 1.94E-04***	3

	transition pathway					
**Glioblastoma multiforme**	GP6 signaling pathway	***1.42E-04***	2.2%	Cell death and survival	2.32E-03 - 1.18E-05	4
	Molecular mechanisms of cancer	***2.02E-04***	1.0%	Lipid metabolism	2.32E-03 - 2.64E-05	6
	Intrinsic prothrombin activation pathway	***4.77E-04***	4.8%	Small molecule biochemistry	2.32E-03 - 2.64E-05	6
	UVC-induced MAPK signaling	***5.01E-04***	4.7%	Cell-to-cell signaling and interaction	2.32E-03 - 2.11E-04	6
	UVB-induced MAPK signaling	***1.18E-03***	3.0%	Cellular function and maintenance	2.32E-03 - 2.11E-04	8
